# Methylation of foreign DNA overcomes the restriction barrier of *Flavobacterium psychrophilum* and allows efficient genetic manipulation

**DOI:** 10.1128/aem.01448-24

**Published:** 2025-01-10

**Authors:** Seada Sloboda, Xinwei Ge, Daqing Jiang, Lin Su, Gregory D. Wiens, Carly A. Beveridge, Eric Duchaud, Mark J. McBride, Tatiana Rochat, Yongtao Zhu

**Affiliations:** 1 Department of Biological Sciences, Minnesota State University Mankato 396294 , Mankato, Minnesota, USA; 2 Department of Biosciences and Bioinformatics, School of Science, Xi’an Jiaotong-Liverpool University 122238 , Suzhou, Jiangsu, China; 3 Department of Biological Sciences, University of Wisconsin-Milwaukee 118734 , Milwaukee, Wisconsin, USA; 4 National Center for Cool and Cold Water Aquaculture, Agricultural Research Service, USDA 57691 , Kearneysville, West Virginia, USA; 5 INRAE, UVSQ, VIM, Université Paris-Saclay 27048 , Jouy-en-Josas, France; Indiana University Bloomington, Bloomington, Indiana, USA

**Keywords:** *Flavobacterium psychrophilum*, bacterial cold-water disease, genetic manipulation, restriction-modification systems, DNA methyltransferases, DNA transfer, type IX secretion system

## Abstract

*Flavobacterium psychrophilum* causes bacterial cold-water disease (BCWD) in salmonids and other fish, resulting in substantial economic losses in aquaculture worldwide. The mechanisms *F. psychrophilum* uses to cause disease are poorly understood. Despite considerable effort, most strains of *F. psychrophilum* have resisted attempts at genetic manipulation. *F. psychrophilum* restriction-modification (R-M) systems may contribute to this resistance. Restriction endonucleases (REases) rapidly degrade nonself DNA if it is not properly methylated by their cognate DNA methyltransferases (MTases). We used comparative genomics to show that R-M systems are abundant in *F. psychrophilum* and that strain-specific variations partially align with phylogeny. We identified two critical type II R-M systems, HpaII-like (FpsJI) and ScrFI-like (FpsJVI), that are conserved in most of the sequenced strains. Protection of foreign DNA against HpaII and ScrFI was achieved by expression of the MTases M.FpsJI and M.FpsJVI in *Escherichia coli*. Furthermore, deleting the two REase genes from *F. psychrophilum* resulted in efficient conjugative DNA transfer from *E. coli* into the otherwise genetically intractable *F. psychrophilum* strain CSF259-93. This allowed us to construct a CSF259-93 mutant lacking *gldN*, a core component of the type IX protein secretion system. The pre-methylation system developed in this study functions in all tested *F. psychrophilum* strains harboring HpaII-like and ScrFI-like REases. These newly developed genetic tools may allow the identification of key virulence factors and facilitate the development of live attenuated vaccines or other measures to control BCWD.

**IMPORTANCE:**

Bacterial cold-water disease (BCWD) caused by *Flavobacterium psychrophilum* is a problem for salmonid aquaculture worldwide, and current control measures are inadequate. An obstacle in understanding and controlling BCWD is that most *F. psychrophilum* strains resist DNA transfer, thus limiting genetic studies of their virulence mechanisms. *F. psychrophilum* restriction enzymes that destroy foreign DNA were suspected to contribute to this problem. Here, we used *F. psychrophilum* DNA methyltransferases to modify and protect foreign DNA from digestion. This allowed efficient conjugative DNA transfer into nine diverse *F. psychrophilum* strains that had previously resisted DNA transfer. Using this approach, we constructed a gene deletion mutant that failed to cause disease in rainbow trout. Further genetic studies could help determine the molecular factors involved in pathogenesis and may aid development of innovative BCWD control strategies.

## INTRODUCTION

The fish pathogen *Flavobacterium psychrophilum*, a member of the phylum *Bacteroidota* (1), is responsible for economic losses in freshwater salmonid aquaculture worldwide (2, 3). *F. psychrophilum* causes bacterial cold-water disease (BCWD), also sometimes referred to as rainbow trout fry syndrome, peduncle disease, and saddleback disease (1, 4–6). Farmed salmonids such as rainbow trout (*Oncorhynchus mykiss*) and coho salmon (*Oncorhynchus kisutch*) are particularly susceptible to this disease (7–9). Non-salmonid fish including Ayu (*Plecoglossus altivelis*), European eel (*Anguilla anguilla*), and common carp (*Cyprinus carpio*) are also impacted by *F. psychrophilum* (10–12). This pathogen infects fish of all ages but is most devastating to fry and fingerlings (13, 14). Infected fish manifest signs such as skin ulcerations, erosion of the caudal peduncle, necrotic lesions near the dorsal fin, epidermal hyperplasia, and splenic hypertrophy (15, 16). In young fish, the disease often occurs as septicemia with high mortalities (up to 70%–90%) and limited outward signs prior to death (7, 16, 17).

Since the original isolation of *F. psychrophilum* in 1948 from diseased coho salmon in the United States (18), numerous strains have been recovered from diseased fish in Asia, Europe, North America, Oceania, and South America (2, 13, 19, 20). *F. psychrophilum* is sometimes present in skin mucus, fins, gills, and eggs of salmonids without gross signs of disease (2, 13, 21), and the disease often manifests under deteriorated conditions such as stress or injury. By genotyping with multilocus sequence typing (MLST) analysis, *F. psychrophilum* strains were grouped into different clonal complexes (CCs) and sequence types (STs), with marked association with the host fish species (22–24).

Currently, the control of BCWD outbreaks in fish farms mainly relies on the use of antibiotics, which raises worries regarding the development of antibiotic-resistant strains (25, 26) and also environmental concerns. Alternative strategies to combat BCWD such as vaccination, bacteriophage therapy (27–29), breeding of resistant fish lines (30–32), warm water treatment (33), and use of probiotics (34) have also been explored or are in use. Vaccination is an attractive approach and has been the focus of many studies (35–40). However, licensed vaccine(s) against BCWD are not yet commercially available in most countries (3, 32).

Genetic manipulation of *F. psychrophilum* strains has been problematic, hampering our ability to better understand host-pathogen interactions as a step toward improved control measures. The only strain feasible for genetic manipulation is OSU THCO2-90, originally isolated from coho salmon and belonging to clonal complex-sequence type 9 (CC-ST9) (41–43). Transposon mutagenesis (39), target gene disruption (44), and markerless gene deletion strategies (17, 45, 46) were used to identify potential virulence genes in this strain. The recently adapted *sacB*-mediated gene deletion strategy (47) makes it feasible to construct genetically stable and unmarked mutants in OSU THCO2-90. Using this method, the roles of the T9SS machinery and the TonB-dependent heme receptor-encoding genes *hfpR* and *bfpR* as virulence factors were characterized (17, 46). Genetic manipulation in other strains has been unsuccessful. This is problematic because MLST analysis indicates that the strain that can currently be genetically manipulated (OSU THCO2-90) is not closely related to CC-ST10, isolates of which are commonly associated with severe outbreaks in farmed rainbow trout worldwide (19, 22, 42, 48–52). Moreover, identifying bacterial determinants mediating host species specificity requires genetic studies across various *F. psychrophilum* genotypes.

A pioneering study discovered that many strains of *F. psychrophilum* were resistant to DNA transfer, possibly due to the existence of restriction-modification (R-M) systems (53), which are known to attack foreign DNA (54, 55). R-M systems are diverse and widely distributed among prokaryotes. They are a first line of defense against foreign DNA and contribute to maintaining genome integrity by differentiating between self and nonself DNA through the action of two enzymatic activities: a restriction endonuclease (REase) and a methyltransferase (MTase). Four major types (I, II, III, and IV) have been identified based on their subunit compositions, recognition sequences, cleavage sites, and cofactor requirements (56). Typical R-M systems (types I–III) each have a REase that recognizes a particular DNA sequence and cuts the DNA and a cognate MTase that methylates specific adenine or cytosine residues to protect the site from cleavage (55). Foreign DNA lacking proper methylation in specific R-M recognition motifs may be destroyed by the cognate REases when it enters a cell, whereas the methylated DNA of the producer strain is protected (Type IV R-M systems are different in that their REases only cleave DNA that has been methylated (57) and thus are not discussed further here).

The restriction barrier caused by the R-M systems can be overcome to improve the efficiency of foreign DNA transfer. Methylation of DNA in an *Escherichia coli* strain expressing cyanobacterial MTase genes increased the efficiency of DNA transfer into specific cyanobacteria by transformation or conjugation (58–60). Similar strategies were used to increase the conjugation frequencies in *Clostridium difficile* (61) and *Neisseria gonorrhoeae* (62). An alternative method, involving the systematic elimination of R-M target motifs from foreign DNA, was successfully applied to *Staphylococcus aureus* (63).

In this study, we developed a pre-methylation system that allows efficient conjugative plasmid transfer from *E. coli* into *F. psychrophilum* CSF259-93, a virulent strain isolated from rainbow trout (64, 65). CSF259-93 has been one of the most studied *F. psychrophilum* strains and is a model system for lipopolysaccharide characterization, proteomics, genomics, genetic selection, and vaccine development (37, 65–67). Whole genome analyses (42) indicate that *F. psychrophilum* CSF259-93 belongs to CC-ST10 and is representative of strains that commonly cause outbreaks in rainbow trout aquaculture systems. Like most other *F. psychrophilum* strains examined, strain CSF259-93 resisted previous attempts at genetic engineering (68, unpublished results). The improvement in DNA transfer described here allowed deletion of a core component of the type IX secretion system (*gldN*) in strain CSF259-93, which resulted in loss of virulence. The pre-methylation system also functioned in other diverse *F. psychrophilum* strains. Genetic manipulation of these bacteria will be useful for studies of host-pathogen interactions. It may help determine the roles and relative importance of virulence factors and facilitate the development of BCWD control measures.

## RESULTS

### R-M systems are widespread and diverse among *F. psychrophilum* strains

**Fig 1:**
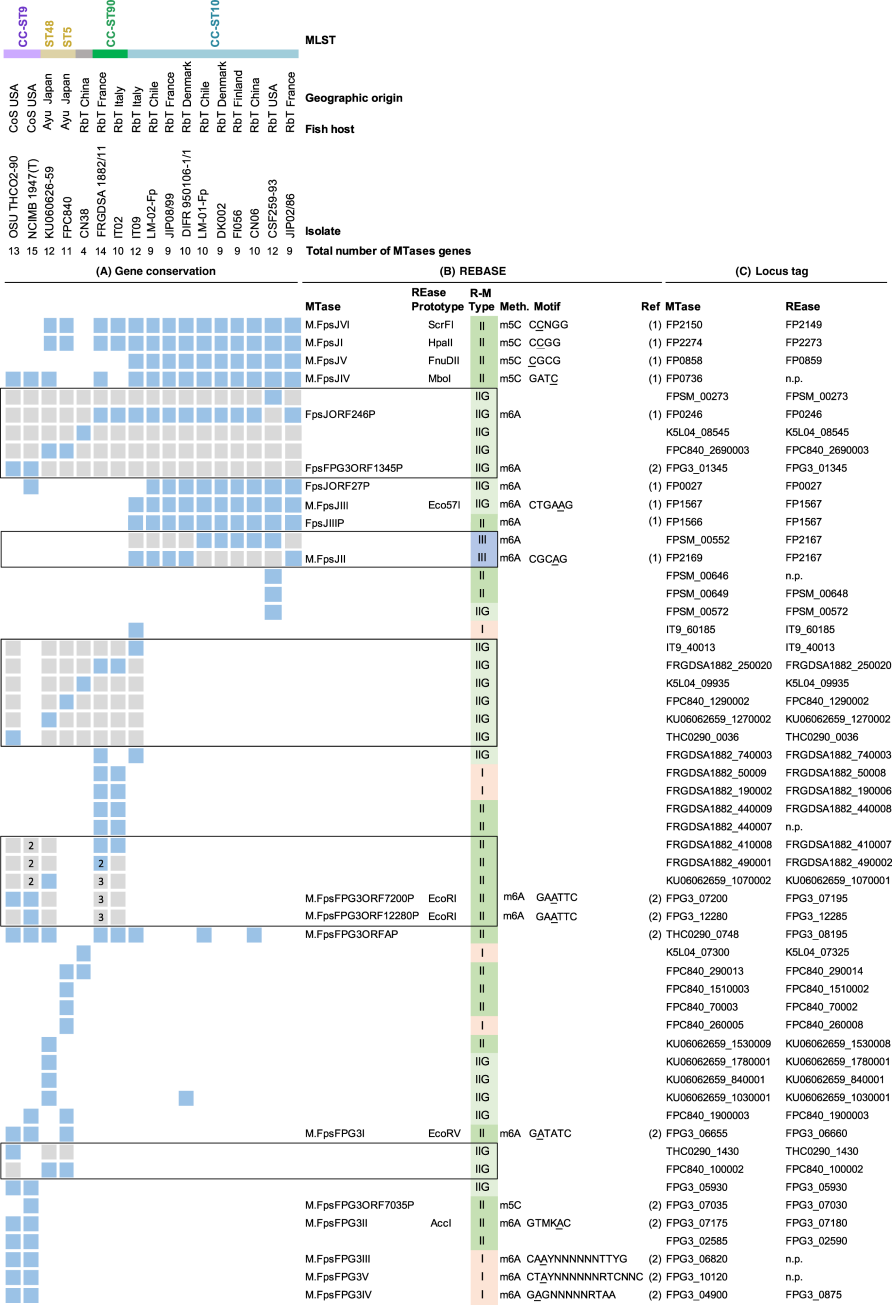
Distribution of MTase-encoding genes in *F. psychrophilum* genomes. (**A**) Conservation of MTase-encoding genes of R-M systems in a collection of 17 strains retrieved from various fish species and geographic origins: blue, orthologous gene present (>90% identity in protein sequence); grey, genes sharing between 50% and 90% identity in protein sequence (each group of homologs is surrounded by a black line); white, gene not conserved (<50% identity in protein sequence). The numbers “2” and “3” inside some squares indicate the number of gene copies present in the genome. All other squares (with no number) indicate that a single copy is present. MLST, multi-locus sequence type data from *F. psychrophilum* PubMLST database (71); CC-ST, clonal complex-sequence type. Fish host: RbT, *Oncorhynchus mykiss*; Ayu, *Plecoglossus altivelis*; CoS, *Oncorhynchus kitsutch*. MLST data are not available for CN38. (**B**) R-M system details from the REBASE database (69): R-M type, MTase name, enzymatic activity (methylated bases and motif specificity), and corresponding REase prototype for isoschizomers. Information is provided for enzymes with demonstrated activity based on PacBio DNA methylation data, available respectively for strains JIP02/86 and NCIMB 1947(T) in references 72 and 73. (**C**) Locus tag of R-M system genes: MTase, DNA methyltransferase encoding gene; REase, restriction endonuclease encoding gene; n.p., gene not present.

Distribution of R-M systems was analyzed by comparative genomics for a collection of 17 *F*. *psychrophilum* strains representative of various fish host species (*O. mykiss*, *O. kitsutch*, and *P. altivelis*), geographic origins (Chile, China, Denmark, Finland, France, Japan, Italy, and the United States), and MLST genotypes (Fig. 1). A total of 55 MTase-encoding genes were identified based on prediction of DNA methyltransferase functional domains (69, 70), and 50 were each associated with a REase. By comparing the sequences of the 55 MTases with each other, some showed substantial homology (>50% of protein sequence identity), suggesting a possible shared enzymatic specificity. Each strain encoded an average of ~10 MTases. Some of these R-M systems were common among many *F. psychrophilum* strains, whereas others had a more narrow distribution. HpaII-like (FpsJI) and ScrFI-like (FpsJVI) were the two most prevalent R-M systems in the analyzed strains. Distribution of R-M systems mainly aligned with clonal complexes, indicating that phylogenetically close strains often had similar R-M systems (Fig. 1; Fig. S1). Strikingly, strains belonging to CC-ST10 harbored six R-M systems that are absent in the genetically tractable strain OSU THCO2-90 (Fig. 1) and may be responsible for the restriction barrier.

PacBio DNA methylation profiles previously established for strains JIP02/86 and NCIMB 1947 (belonging, respectively, to CC-ST10 and -ST9) were retrieved from the New England Biolabs REBASE (69, 72, 73). These experimental data allowed assignment of recognition sequences and methylated bases for six of nine JIP02/86 R-M systems and for eight of 15 NCIMB 1947 R-M systems (Fig. 1). DNA methylation by M.FpsJIV, an orphan MTase, was detected by PacBio in GATC motifs despite the apparent absence of a cognate REase (MboI prototype). These results indicate that a majority of encoded R-M systems are functional in these *F. psychrophilum* strains. For clarity, we will refer to the *F. psychrophilum* REases by the names of their corresponding REBASE prototypes.

### Multiple R-M systems are present in *F. psychrophilum* strain CSF259-93

In order to further identify the R-M systems likely responsible for the restriction barrier in strains associated with BCWD outbreaks in rainbow trout, we performed an in-depth analysis of *F. psychrophilum* CSF259-93. The *F. psychrophilum* CSF259-93 genome encodes 12 R-M systems (Table 1). Eleven of these are type II R-M systems, with four belonging to the type IIG subgroup, which generally have both MTase and REase activities in a single polypeptide (57). The recognition sequences were predicted for nine of the R-M systems based on PacBio data or sequence similarity with prototype enzymes. No REase was identified for two out of the 12 MTases, including M.FpsJIV that is highly prevalent in analyzed strains, indicating that those two may be orphan MTases, not in active R-M systems.

**TABLE 1:** Predicted R-M systems in *F. psychrophilum* CSF259-93^*a*^

R-M type	Function^*b*^	Name (REBASE)^*c*^	REase prototype^*d*^	*F. psychrophilum* MTase name	NCBI locus tag^*c*^	Recognition sequence (RS)^*e*^	Number of RSs on pCP11/pYT313
II	R	Fps93ORF2394P	HpaII		FPSM_02393	CCGG	23/22
	M	M.Fps93ORF2394P		M.FpsJI	FPSM_02394		
II	R	Fps93ORF612P	ScrFI		FPSM_00613	CCNGG (N = A/T/G/C)	22/20
	M	M.Fps93ORF612P		M.FpsJVI	FPSM_00612		
II	R	Fps93ORF649P	TauI^*f*^		FPSM_00648	GCSGC^*f*^ (S = G/C)	19/19
	M	M.Fps93ORF649P		M.FpsJVII^*f*^	FPSM_00649		
II	R	n.p.	MboI		n.p.	GATC	17/21
	M	M.Fps93ORF1581P		M.FpsJIV	FPSM_01581		
II	R	Fps93ORF1519P	FnuDII		FPSM_01518	CGCG	14/17
	M	M.Fps93ORF1519P		M.FpsJV	FPSM_01519		
II	R	n.p.	Unknown		n.p.	Unknown	Unknown
	M	M.Fps93ORF646P			FPSM_00646		
II	M	M.Fps93ORF1246P		FpsJIIIP^*g*^	FPSM_01247–01248^*h*^	CTGAAG	4/4
IIG	RM	Fps93ORF1246P	Eco57I	M.FpsJIII	FPSM_01246		
IIG	RM	Fps93ORF572P	Jma19592I^*f*^		FPSM_00572	GTATNAC^*f*^	1/1
IIG	RM	Fps93ORF273P	Unknown		FPSM_00273	Unknown	Unknown
IIG	RM	Fps93ORF28P	Unknown	FpsJORF27P	FPSM_00028	Unknown	Unknown
III	R	Fps93IP	FpsJIIP		FPSM_00554	CGCAG	8/8
	M	M.Fps93I		M.FpsJII	FPSM_00552		

^*a*^ R-M systems as predicted by REBASE (http://rebase.neb.com/rebase/rebase.html).

^*b*^ “R” indicates REase, “M” indicates MTase, and “RM” indicates bifunctional enzyme that has both REase and MTase activities.

^*c*^ “n.p.” indicates gene not present in CSF259-93.

^*d*^ The REase prototype is the first enzyme to have been discovered with a particular recognition sequence.

^*e*^ Underline indicates the site of methylation.

^*f*^ Putative enzyme specificity is predicted solely based on sequence similarity with a prototype enzyme (no PacBio DNA methylation data available). The name M.FpsJVII is given to M.Fps93ORF649P in accordance to the REbase nomenclature.

^*g*^ FpsJIIIP probably acts as second MTase of the type IIG Eco57I - M.FpsJIII R-M system due to their operonic organization.

^*h*^ In the NCBI genome sequence of CSF259-93, the 2 ORFs FPSM_01247/FPSM_01248 result from a frameshift. We showed by sequencing that this frameshift is a probable assembly artifact in the original genome sequence and that a full-length FP1566 homolog is present in CSF259-93.

We searched two plasmids, pCP11 and pYT313, that are commonly used in genetic analysis of *Flavobacterium* species, for recognition sequences predicted to be cleaved by CSF259-93 REases. pCP11 is an *E. coli–Flavobacterium* shuttle vector (74), and pYT313 is a suicide vector carrying the *sacB* gene used to construct deletions and other targeted chromosomal modifications in some *Bacteroidota* species (47). Nine of the CSF259-93 R-M systems were predicted to target these plasmids (Table 1). These R-M systems may thus be important restriction barriers that prevent transfer of pCP11, pYT313, or related plasmids into CSF259-93. Two REases, FpsJI and FpsJVI, hereafter also referred to as Fps.HpaII and Fps.ScrFI, respectively (to match their prototypes), were predicted to cut pCP11 or pYT313 at many sites. There were 23 HpaII and 22 ScrFI recognition sequences on pCP11 and 22 HpaII and 20 ScrFI recognition sequences on pYT313.

### Pre-methylation of pCP11 by M.FpsJI and M.FpsJVI overcame the restriction barrier and allowed successful DNA transfer into CSF259-93

**Fig 2:**
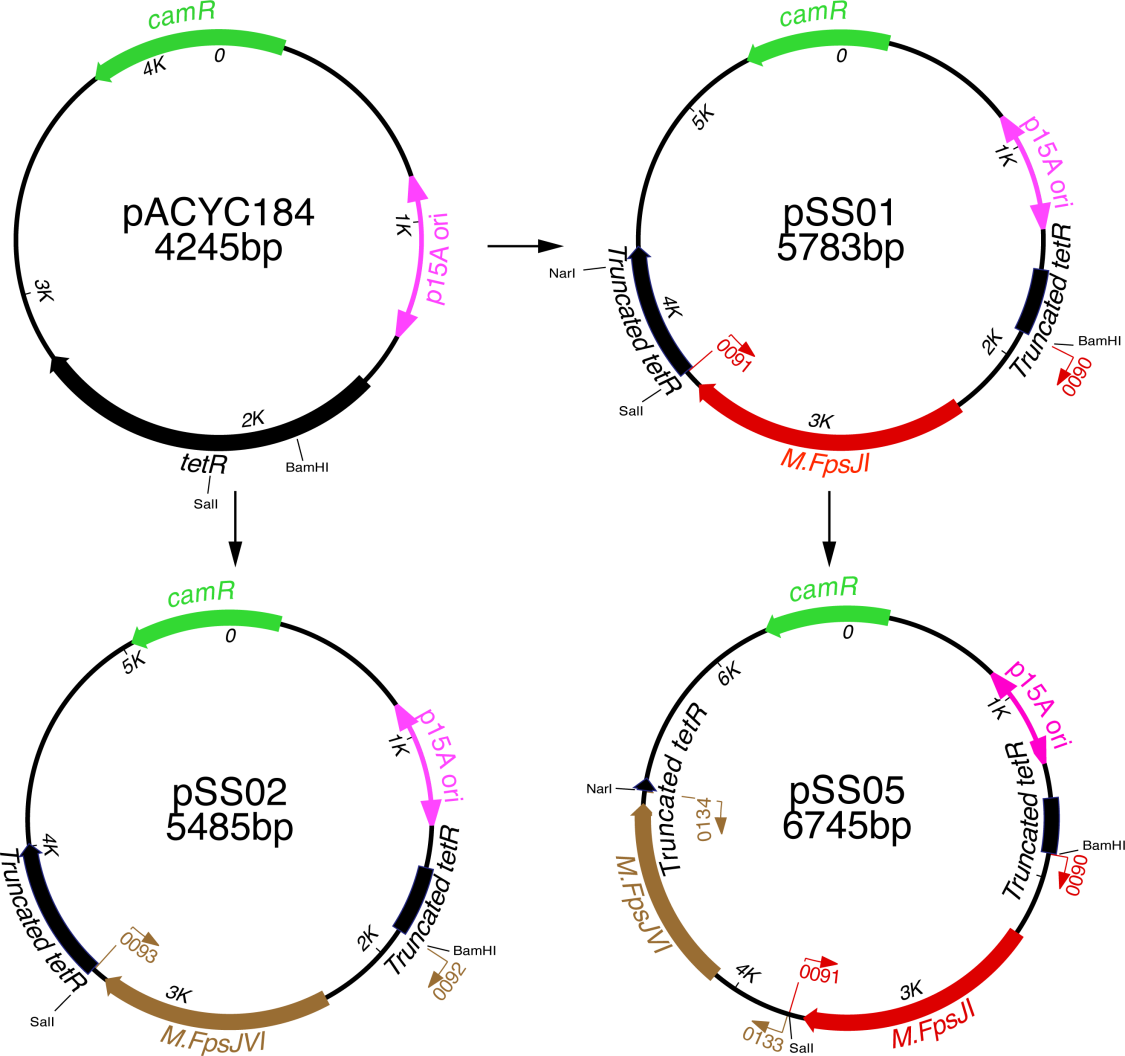
Maps of pACYC184 and the representative methylation plasmids. M.FpsJI and M.FpsJVI-encoding genes were cloned between the BamHI and SalI sites of pACYC184 to generate pSS01 and pSS02, respectively. M.FpsJVI gene was cloned between the SalI and NarI sites of pSS01 to generate pSS05. Other methylation plasmids listed in Table S1 (pSS07, pSS08, pSS11, and pCB01) were constructed by inserting the corresponding MTase gene between the BamHI and NarI sites of pSS01. Numbers immediately inside of the ring refer to base pairs of sequence. p15A *ori* refers to the origin of replication that functions in *E. coli*, but not in *F. psychrophilum. camR* and *tetR* confer chloramphenicol and tetracycline resistance on *E. coli*, respectively, but not on *F. psychrophilum*. Binding sites for primers used in PCR reactions to clone the MTase genes are shown by the arrows inside and outside of the rings, with the perpendicular ends indicating the actual binding sites.

We cloned each of the seven genes encoding CSF259-93 MTases M.FpsJI (FPSM_02394), M.FpsJVI (FPSM_00612), M.FpsJIV (FPSM_01581), M.FpsJII (FPSM_00552), M.FpsJVII (FPSM_00649), M.FpsJV (FPSM_01519), and M.FpsJIII (FPSM_01246) into the moderate copy-number vector pACYC184 (75–77) to generate methylation plasmids pSS01, pSS02, pSS03, pSS07, pSS08, pSS11, and pCB01, respectively (Fig. 2; Table S1). pACYC184 has the p15A *ori* and is compatible with plasmids carrying the pMB1 *ori*, including pCP11 and pYT313. Each cloned MTase-encoding gene retains its putative *F. psychrophilum* promoter, and they were inserted downstream of the tetracycline resistance gene (*tetR*) promoter on pACYC184 (Fig. 2). Each methylation plasmid was co-transformed with pCP11 into the conjugation donor strain *E. coli* S17-1 λ *pir* (78). Expression of the MTase in the donor *E. coli* cells was expected to methylate the corresponding recognition motifs on pCP11 and protect it from digestion by the cognate *F. psychrophilum* REase. The efficiency of stable transfer of pCP11 from *E. coli* S17-1 λ *pir* to *F. psychrophilum* CSF259-93 and its replication was determined by the growth of erythromycin-resistant colonies after conjugation since pCP11 confers erythromycin resistance to *F. psychrophilum* (46).

**Fig 3:**
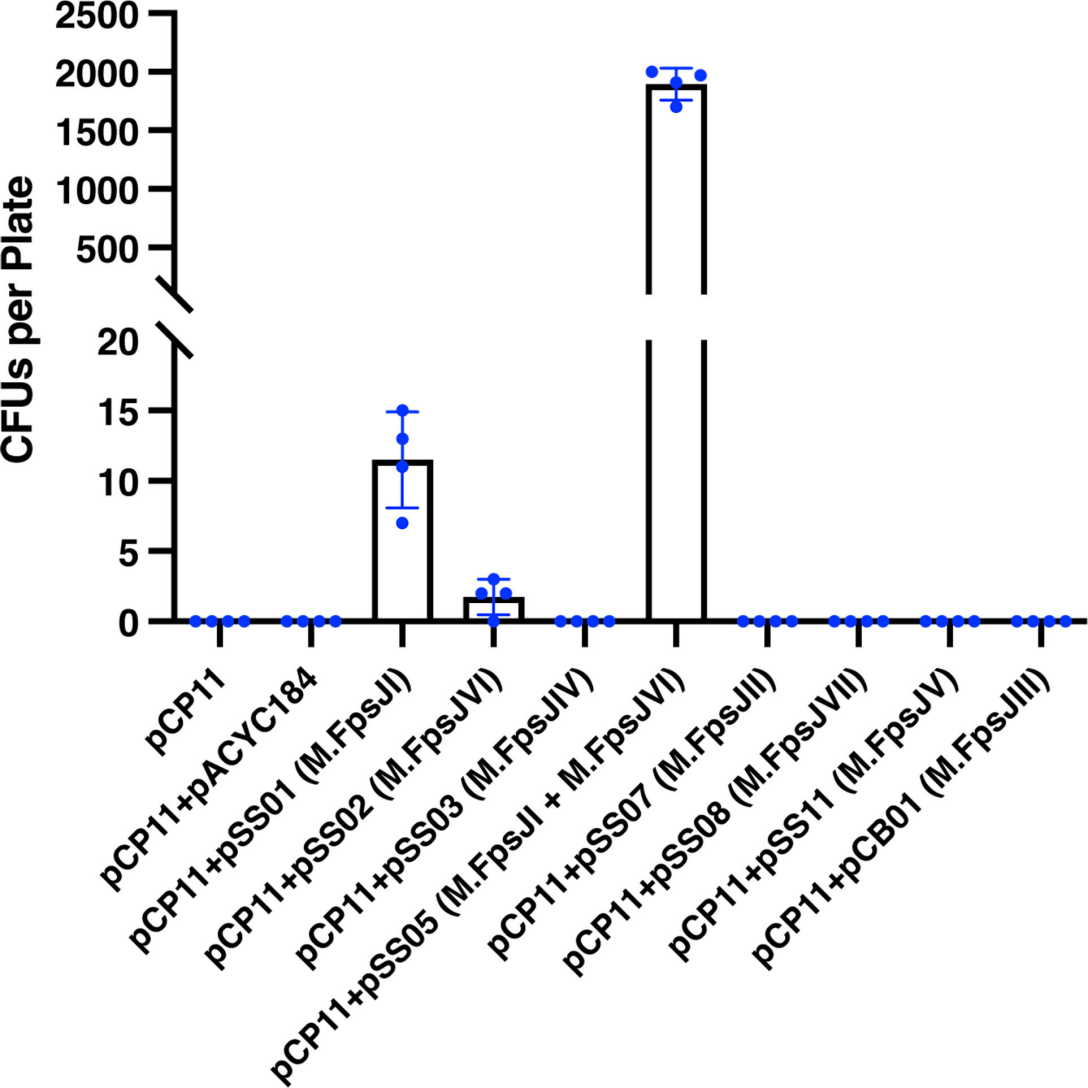
Improvement of conjugation efficiency in *F. psychrophilum* CSF259-93 using different methylation plasmids. The *E. coli* S17-1 λ *pir* strains, carrying both pCP11 and one of the different methylation plasmids, were used as the donor, and wild-type *F. psychrophilum* CSF259-93 was used as the recipient in the conjugations. *E. coli* S17-1 λ *pir* carrying pCP11 and pACYC184 was used as a negative control. Equal amounts of *F. psychrophilum* and *E. coli* cells were used in all the conjugation experiments. Erythromycin-resistant CFUs were counted from four plates in each conjugation experiment. Error bars represent standard deviation.

Without the protection provided by MTase-bearing plasmids, conjugation from *E. coli* carrying pCP11 only, or carrying pCP11 plus pACYC184, was unsuccessful, and no erythromycin-resistant colonies were obtained (Fig. 3). The plasmids that carried M.FpsJII, M.FpsJIII, M.FpsJIV, M.FpsJV, and M.FpsJVII failed to result in transconjugants, presumably because they did not protect pCP11 from the critical *F. psychrophilum* restriction enzymes. In contrast, pSS01 and pSS02, carrying M.FpsJI and M.FpsJVI, respectively, allowed the growth of 2–15 erythromycin-resistant colonies on each TYES agar plate after conjugation (Fig. 3). We confirmed the presence of pCP11 in these colonies by PCR (data not shown). pSS05, containing both M.FpsJI and M.FpsJVI, was further constructed and found to increase the number of erythromycin-resistant colonies on each plate by approximately two orders of magnitude to 1895 ± 135 (Fig. 3), suggesting that protection against both Fps.HpaII and Fps.ScrFI allowed efficient stable transfer of pCP11. pSS05 also protected pCP23, another *E. coli–F. psychrophilum* shuttle vector (53, 79), and allowed its conjugative transfer with similar efficiency (2760 ± 46 CFUs / plate).

### The methylation plasmid pSS05 protects pCP11 from restriction digestion *in vitro*

**Fig 4:**
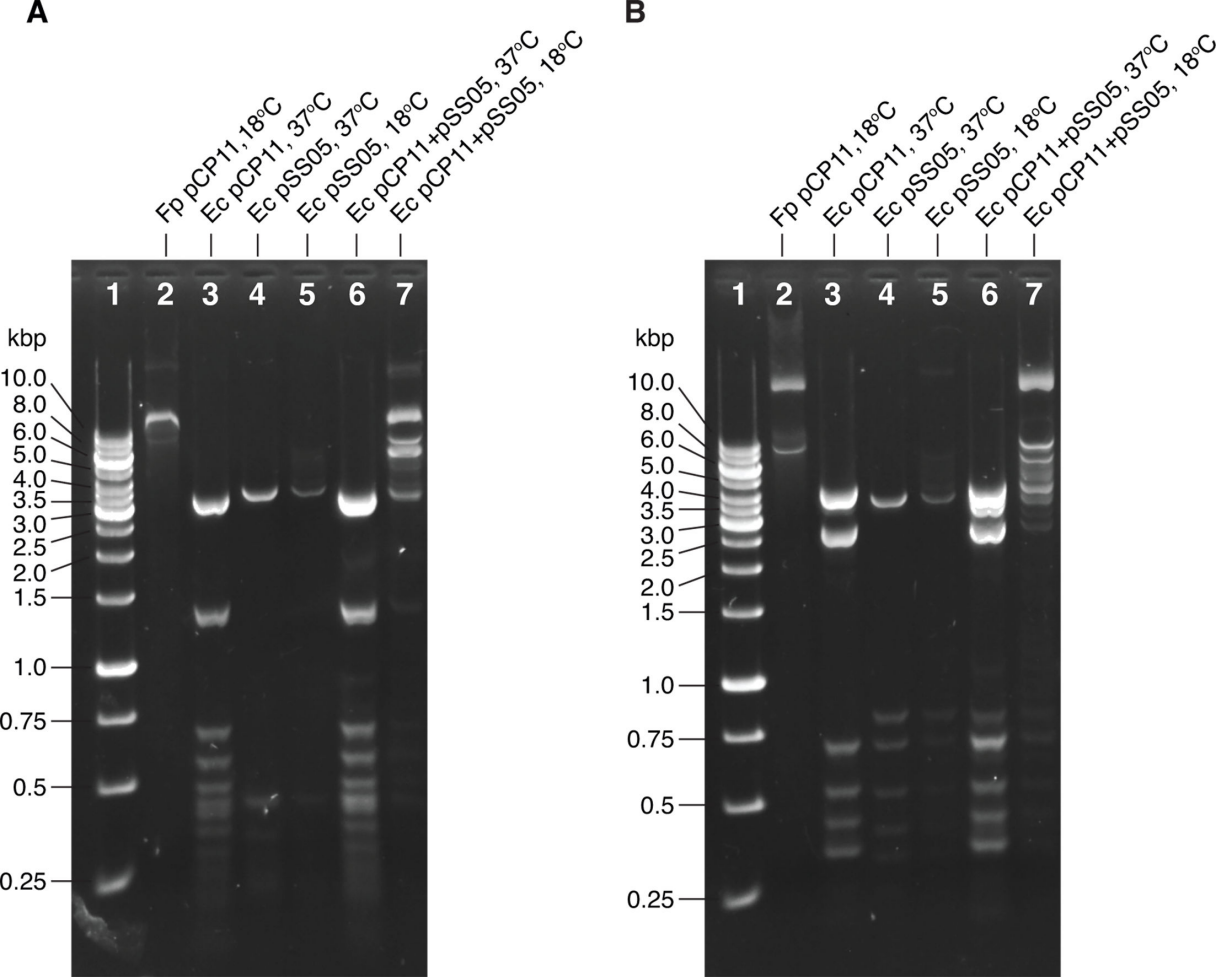
*In vitro* restriction digestion by HpaII (**A**) or ScrFI (**B**) to verify the pre-methylation-mediated protection of pCP11. The digestion pattern of properly methylated pCP11 isolated from the wild-type *F. psychrophilum* CSF259-93 (grown at 18°C) was used as a control to show the full protection of methylation (lane 2). Lane 1 was loaded with the DNA ladder (Thermo Scientific, SM1163). Lanes 3–7 used plasmids isolated from *E. coli* S17-1 λ *pir* grown at different temperatures. The *E. coli* strains contained pCP11 only, pSS05 only, or pCP11 plus pSS05 were cultivated at 37°C or 18°C as indicated. Samples in panel A were digested by HpaII and in B digested by ScrFI. Intact pCP11 is 9448 bp, and pSS05 is 6745 bp. “Fp” indicates *F. psychrophilum* CSF259-93. “Ec” indicates *E. coli* S17-1 λ *pir*.

To verify that pCP11 was modified by expression of the MTase genes carried on pSS05, we isolated pCP11 from *E. coli* S17-1 λ *pir* with or without the presence of pSS05 and performed *in vitro* restriction digestions using either HpaII or ScrFI. As expected, pCP11 isolated from *F. psychrophilum* CSF259-93 was substantially protected from digestion by these enzymes, whereas pCP11 isolated from *E. coli* in absence of pSS05 was digested into small fragments (Fig. 4). Interestingly, pCP11 isolated from *E. coli* carrying pSS05 grown at 37°C was readily digested by HpaII or ScrFI. However, when pCP11 was isolated from the same strain that was cultured at 18°C, a temperature preferred by the psychrotrophic *F. psychrophilum*, it was resistant to digestion by both enzymes (Fig. 4).

### pSS05 facilitated the deletion of Fps.HpaII, Fps.ScrFI, and GldN encoding genes in *F. psychrophilum* CSF259-93

A *sacB*-mediated gene deletion system tailored to *Bacteroidota* was recently developed (47). This system involves the suicide vector pYT313-mediated DNA recombination and has been used to make mutations in *F. psychrophilum* OSU THCO2-90 (17, 46). There are 22 HpaII and 20 ScrFI recognition motifs on the pYT313 sequence (Table 1), suggesting that deletion constructs derived from this plasmid would require methylation by pSS05 before transfer to *F. psychrophilum* CSF259-93. To test the ability of pSS05 to allow gene deletion using pYT313, we constructed pXG01, pXG02, and pSS12 (Table S1) to delete genes encoding Fps.ScrFI (FPSM_00613), Fps.HpaII (FPSM_02393), and GldN (FPSM_00826), respectively. GldN is one of the core components in the type IX secretion system (T9SS), known to be essential for gliding motility and for virulence of *F. psychrophilum* OSU THCO2-90 (46).

pXG01, pXG02, and pSS12 were each successfully transferred to *F. psychrophilum* CSF259-93 by conjugation from *E. coli* S17-1 λ *pir* that also carried the methylation plasmid pSS05. Approximately 3–10 colonies were obtained on each erythromycin plate. The resulting colonies had pXG01, pXG02, or pSS12 integrated into the chromosome by homologous recombination. Selection for loss of the plasmid by exposure to sucrose identified mutants where a second recombination event had resulted in deletion of the Fps.ScrFI, Fps.HpaII, or GldN encoding gene (Fig. S2). This demonstrates the usefulness of this approach to allow construction of targeted mutations in *F. psychrophilum* strains such as CSF259-93.

### Elimination of the Fps.HpaII and Fps.ScrFI REases resulted in highly efficient stable DNA transfer

**Fig 5:**
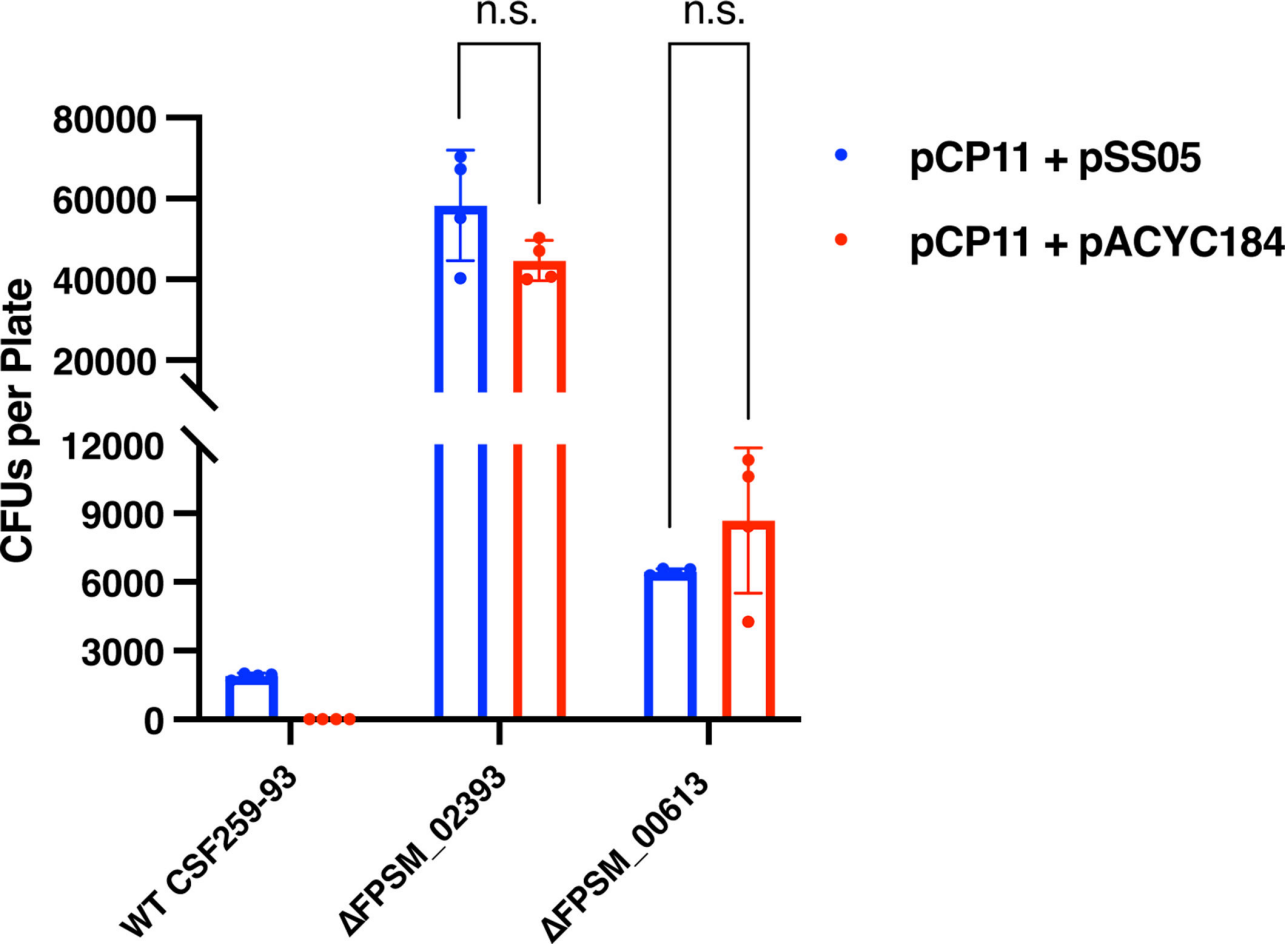
Elimination of the Fps.HpaII and Fps.ScrFI REases in *F. psychrophilum* CSF259-93 resulted in increased conjugation efficiency. The *E. coli* S17-1 λ *pir* strains carrying pCP11 plus pSS05 or pCP11 plus pACYC184 were used as the donor, and wild-type *F. psychrophilum* CSF259-93 or mutants lacking Fps.HpaII (∆FPSM_02393) or Fps.ScrFI (∆FPSM_00613) were used as recipients in the conjugations. Equal amounts of *F. psychrophilum* and *E. coli* cells were used in all the conjugation experiments. Erythromycin-resistant CFUs were counted from four plates in each conjugation experiment. Comparison of conjugation efficiencies was performed by t test using GraphPad Prism v10.2.3. Error bars represent standard deviation.

Conjugation of pCP11 into wild-type *F. psychrophilum* CSF259-93 and into mutants lacking Fps.HpaII or Fps.ScrFI was examined. As shown in Fig. 5, conjugation into the wild type failed unless a suitable methylation plasmid (here pSS05) was present. In contrast, pCP11 was efficiently transferred into the mutants, with 4.4 ± 0.5 × 10^4^ and 8.7 ± 3.2 × 10^3^ CFUs per plate obtained for the Fps.HpaII and Fps.ScrFI mutants, respectively. These numbers were even higher than those obtained for conjugation of pCP11 from *E. coli* carrying the pre-methylation plasmid pSS05 into wild-type *F. psychrophilum* CSF259-93 (Fig. 5). The presence of pSS05 in the donor *E. coli* strain did not significantly increase the conjugation efficiencies into the mutants lacking Fps.HpaII or Fps.ScrFI. The results indicate that elimination of either the HpaII or ScrFI-like REase effectively overcame the DNA transfer barrier.

### *gldN* deletion mutant exhibited defects in gliding motility, extracellular proteolytic activity, and virulence

**Fig 6:**
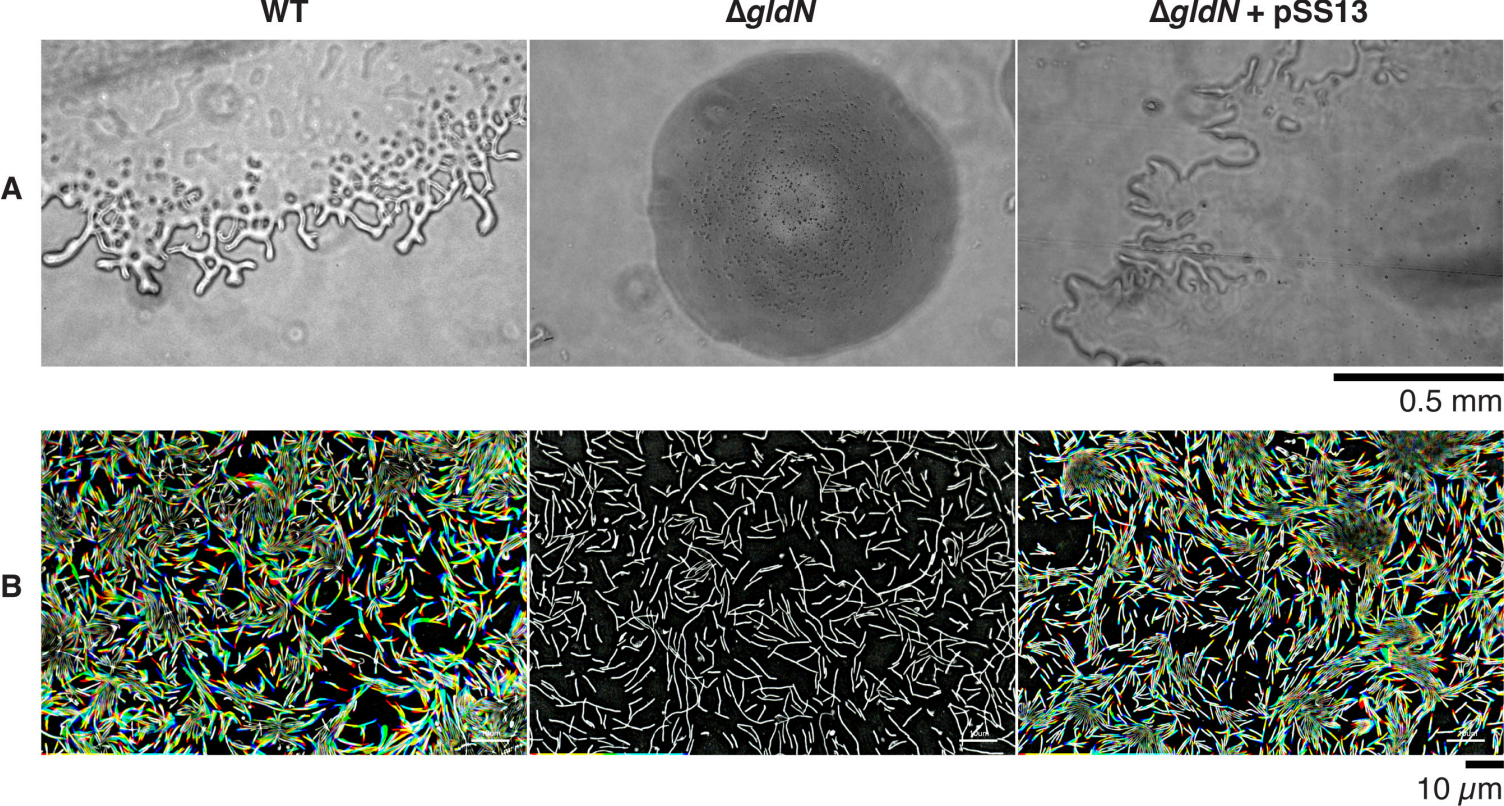
Photomicrographs of *F. psychrophilum* colonies (row A) and gliding motility of individual cells on agar (row B). WT indicates wild-type CSF259-93, ∆*gldN* indicates *gldN* deletion mutant, and ∆*gldN* + pSS13 indicates *gldN* mutant complemented with wild-type *gldN* on pSS13. Colonies grown from single cells were incubated for 8 days at 18°C on 5% TYES solidified with 1% agar (row A). Scale bar beneath row A indicates 0.5 mm and applies to all panels of row A. For individual cell motility assay (row B), cells were grown in TYES at 18°C with shaking for 48 h, spotted on a pad of full-strength TYES solidified with 1% agar on a glass slide, and covered with an O_2_-permeable Teflon membrane. A series of images were taken, and individual frames were colored from red (time 0) to yellow, green, cyan, and finally blue (24 s) and integrated into one image, resulting in “rainbow traces” of gliding cells. White cells correspond to cells that exhibited little if any net movement. The scale bar at lower right indicates 10 µm and applies to all panels of row B.

**Fig 7:**
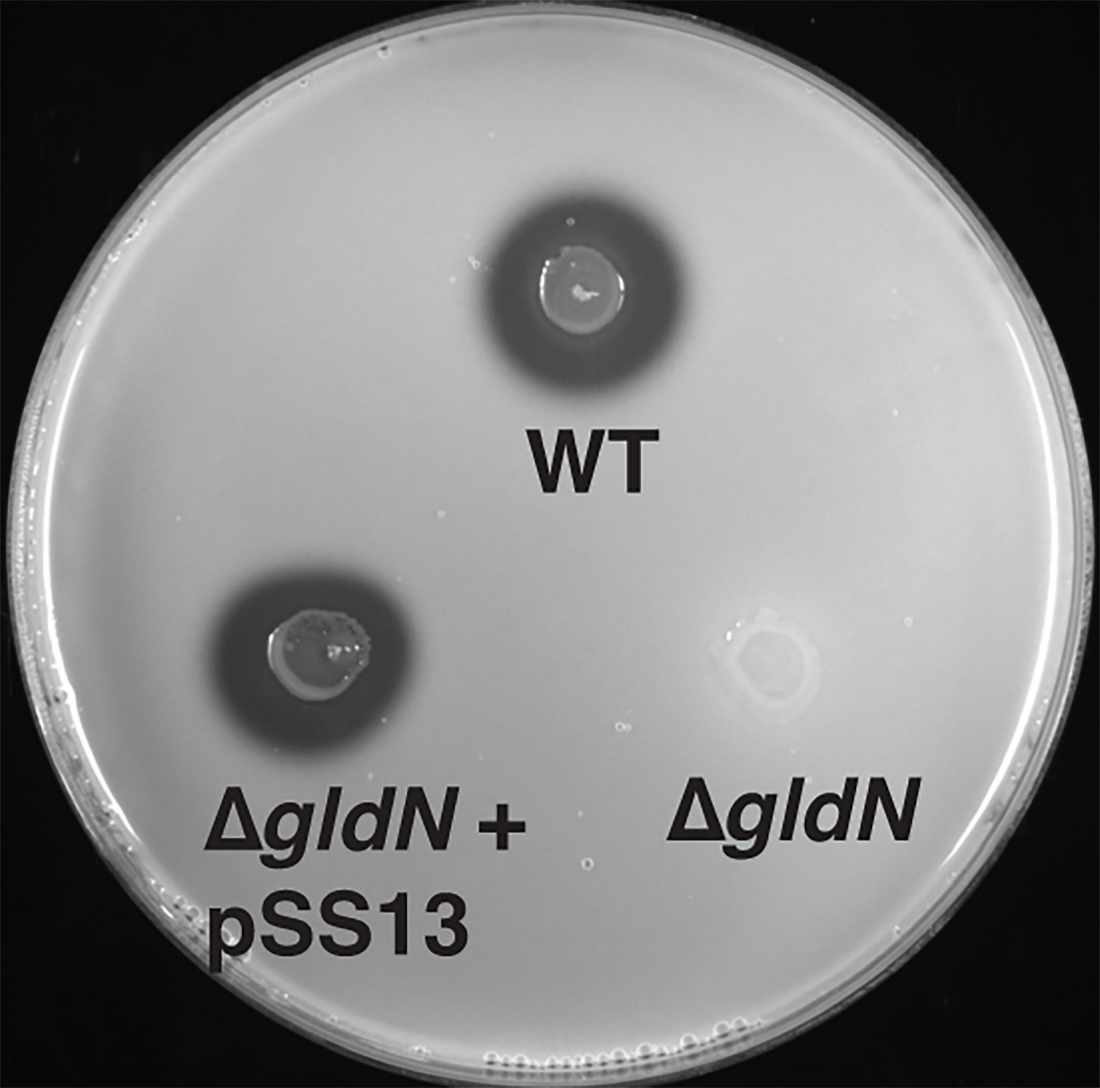
Secreted proteolytic activity of the wild-type *F. psychrophilum* CSF259-93 (WT), *gldN* mutant (∆*gldN*), and complemented strain (∆*gldN* + pSS13). Equal amounts of cells were spotted on TYES supplemented with 1.5% skim milk and incubated at 18℃ for 7 days. Clear zones surrounding the area of growth indicate hydrolysis of skim milk by the secreted enzymes.

Consistent with the previously reported results in *F. psychrophilum* THCO2-90 (46), the CSF259-93 *gldN* mutant failed to spread or move on agar surfaces (Fig. 6). Proteolytic activity analyses showed that the mutant also failed to hydrolyze skim milk proteins (Fig. 7). These results were expected because GldN is a component of the T9SS machinery, which secretes many proteins, including peptidases and adhesins required for gliding motility, in *F. psychrophilum* and other *Bacteroidota* (46, 80, 81). Complementation using pCP11 carrying the wild-type *gldN* gene restored motility and secreted proteolytic activity to the mutant (Fig. 6 and 7).

**Fig 8:**
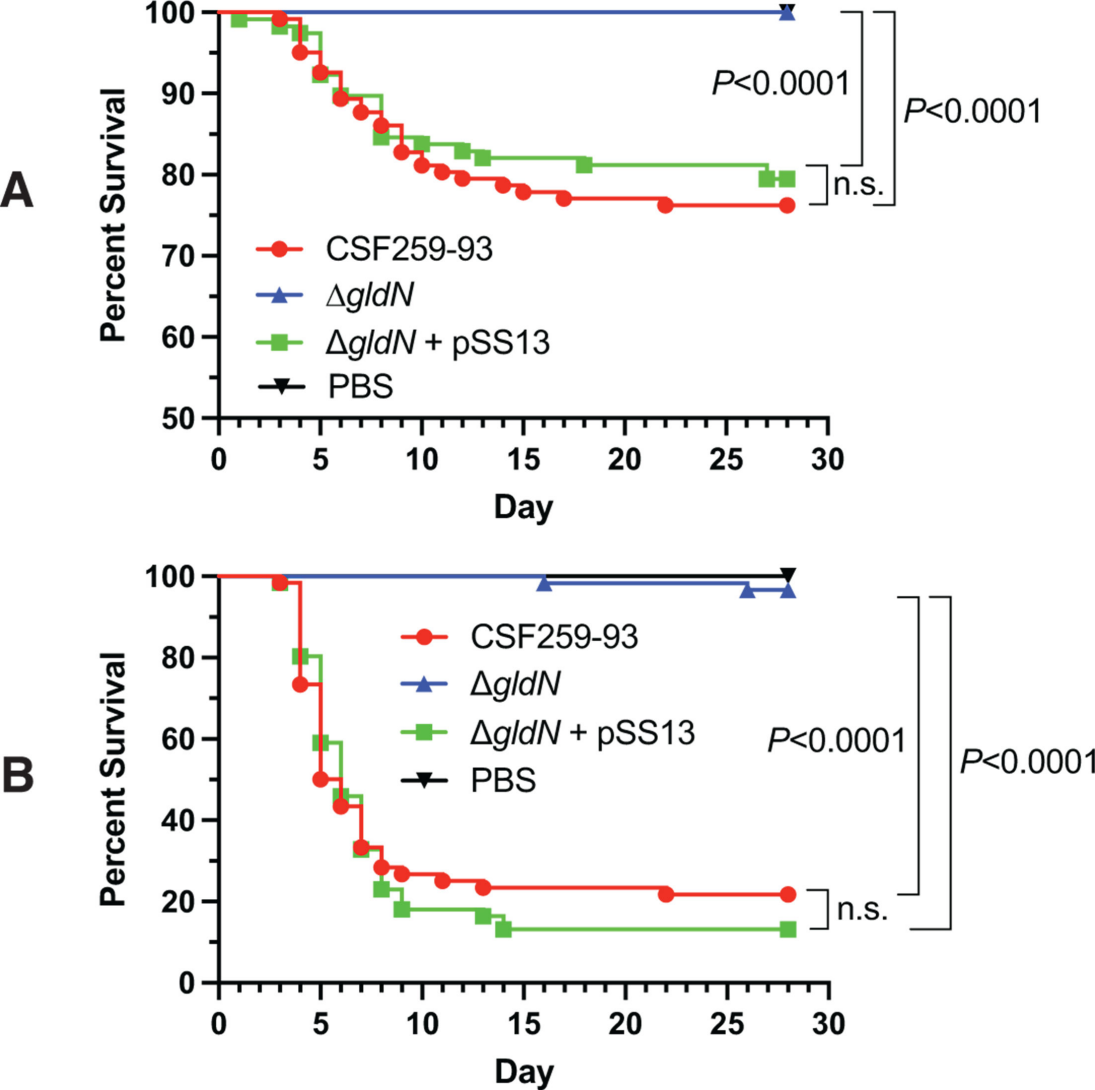
The percent survival of rainbow trout after challenge with *F. psychrophilum*. For panel A (trial 1), each fish (Troutlodge February spawning line) was injected as indicated in Materials and Methods with 20 µL of PBS, wild-type CSF259-93 (9.17 ± 0.32 × 10^6^ CFU), *gldN* mutant (11.1 ± 1.08 × 10^6^ CFU), and the complemented strain (7.57 ± 1.23 × 10^6^ CFU). For panel B (trial 2), each fish (Troutlodge May spawning line) was injected with 50 µL of PBS, wild-type CSF259-93 (17.7 ± 2.57 ×10^6^ CFU), *gldN* mutant (17.8 ± 2.25 × 10^6^ CFU), and the complemented strain (26.7 ± 0.76 × 10^6^ CFU). The percent survival was observed and measured. Comparison of survival curves was performed by Log-rank (Mantel-Cox) test using GraphPad Prism v9.3.1.

Rainbow trout of two spawning lines were challenged with bacterial cells of the wild-type strain CSF259-93, the *gldN* mutant, and the complemented strain. The mutant was nearly completely avirulent in the tested fish, whereas virulence was restored in the complemented strain (Fig. 8). These results indicate that GldN, which is an essential component of the T9SS, is important for virulence of *F. psychrophilum* CSF259-93.

### pSS05 improved the conjugation efficiencies in diverse *F. psychrophilum* strains of different geographic origins

**Fig 9:**
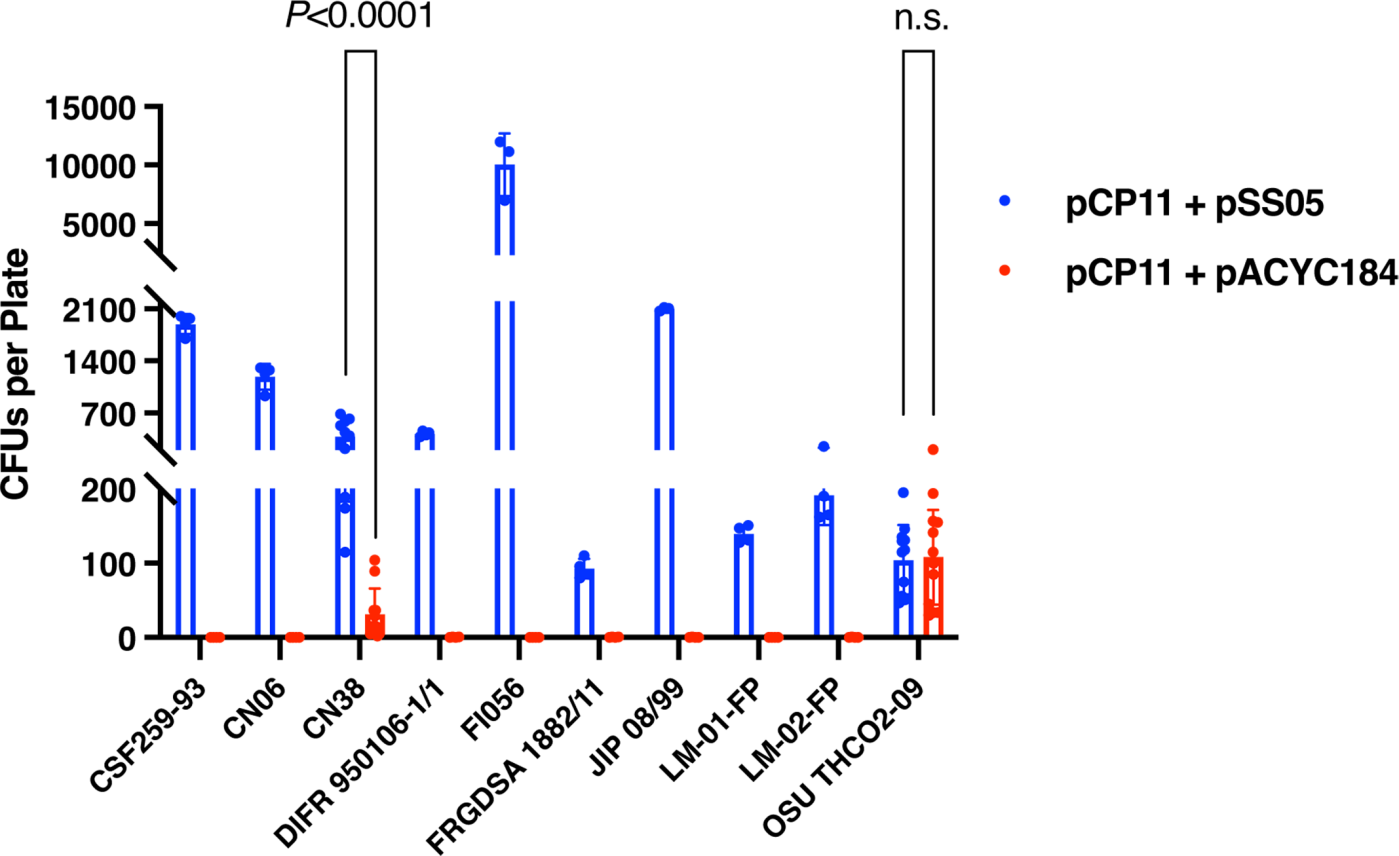
Improvement of conjugation efficiency in different strains of *F. psychrophilum* by the methylation plasmid pSS05. The *E. coli* S17-1 λ *pir* strains carrying pCP11 plus pSS05 or pCP11 plus pACYC184 (negative control) were used as the donors, and wild-type *F. psychrophilum* strains were used as the recipients in the conjugations. Equal amounts of *F. psychrophilum* and *E. coli* cells were used in all the conjugation experiments. Erythromycin-resistant CFUs were typically counted from four plates in each conjugation experiment. Data from a total of 12 plates and three independent experiments were used for CN38 and OSU THCO2-09. Comparison of conjugation efficiencies was performed by t test using GraphPad Prism v10.2.3. Error bars represent standard deviation.

The conjugative transfer efficiency of pCP11 with and without the methylation plasmid pSS05 was determined in nine other *F. psychrophilum* strains, isolated in Chile (LM-01-Fp and LM-02-Fp), China (CN06 and CN38), Denmark (DIFR 950106-1/1), Finland (FI056), France (FRGDSA 1882/11 and JIP08/99), and the United States (OSU THCO2-90) (Fig. 1 and 9). In the absence of pSS05, stable transfer of pCP11 failed for all strains except OSU THCO2-90, which was already known to accept pCP11 without pre-methylation (46), and CN38 (Fig. 9). Approximately 108 ± 64 CFUs per plate were obtained for strain OSU THCO2-90 without pSS05, and 31 ± 35 for strain CN38 without pSS05, indicating the pre-methylation by M.FpsJI or M.FpsJVI was not essential for successful conjugation in these two strains.

Pre-methylation of pCP11 with pSS05 resulted in increased conjugation efficiencies for most strains with ~100–10,000 CFUs obtained per plate (Fig. 9). As expected, pSS05 did not significantly increase the efficiency for OSU THCO2-90 since this strain does not have the HpaII-like and ScrFI-like R-M systems (Fig. 1). Surprisingly, pSS05 significantly increased the efficiency for strain CN38, which also apparently lacks these two R-M systems.

## DISCUSSION

A major obstacle to deciphering the roles of *F. psychrophilum* genes in its physiology and virulence was the lack of efficient genetic manipulation tools in other strains than OSU THCO2-90. Genetic tools were ineffective in the strains responsible for major BCWD outbreaks, impeding experimental investigations into the bacterial determinants driving host association and the development of live attenuated vaccine candidates for relevant epidemic strains.

In this study, we analyzed the genomes of 17 *F. psychrophilum* strains and found that genes encoding R-M systems were widely distributed among them. The HpaII-like and ScrFI-like R-M systems were predicted in strain CSF259-93 and 13 other strains. OSU THCO2-90 is one of the three strains examined that do not have these two R-M systems, and this may explain why genetic manipulation of OSU THCO2-90 using pCP11 and pYT313 was successful (17, 46).

**Fig 10:**
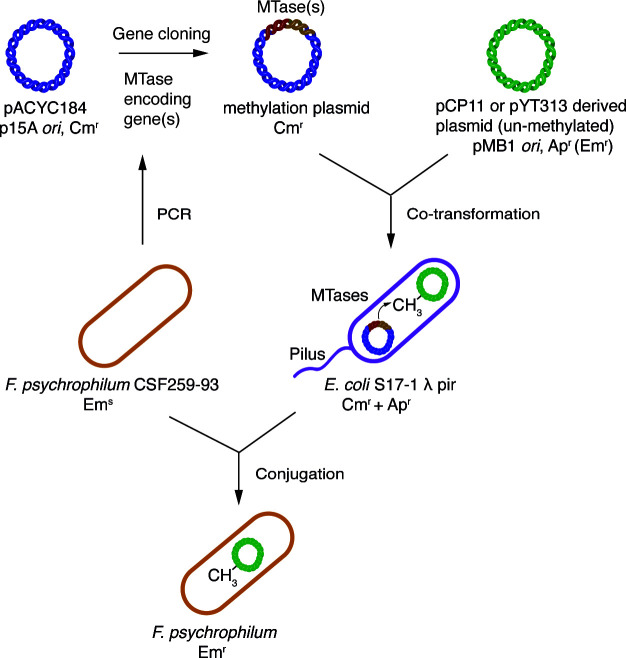
Scheme of the pre-methylation system developed in this study. Ap^r^, ampicillin resistant; Cm^r^, chloramphenicol resistant; Em^r^, erythromycin resistant (expressed in *F. psychrophilum* but not in *E. coli*); and Em^s^, erythromycin sensitive.

We constructed pre-methylation systems using *F. psychrophilum* MTases to bypass the restriction barriers during conjugative DNA transfer (Fig. 10). The plasmid pSS05, which carries *F. psychrophilum* strain CSF259-93 M.FpsJI and M.FpsJVI, protected pCP11- and pYT313-based plasmids during conjugative transfer into this strain. Deletion of the cognate REase (Fps.HpaII or Fps.ScrFI) encoding gene from *F. psychrophilum* strain CSF259-93 similarly eliminated the restriction barrier and allowed high-efficiency DNA transfer. We are not sure why eliminating only one REase had such a significant effect on increasing the DNA transfer efficiency. Probably protection against either Fps.HpaII or Fps.ScrFI allowed the foreign DNA to survive longer after it entered the *F. psychrophilum* cells, giving enough time for MTases to function and provide sufficient protection. Regardless, these results suggest that the REases in the HpaII-like and ScrFI-like R-M systems are major barriers of DNA transfer in *F. psychrophilum* CSF259-93.

The presence of pSS05 in the donor *E. coli* also significantly improved the efficiencies of conjugation of pCP11 into other strains isolated from different geographic locations, indicating that overcoming the restriction barriers of HpaII-like and ScrFI-like R-M systems allows successful DNA transfer in many diverse *F. psychrophilum* strains. With the exception of CN38, each of these has genes predicted to encode the HpaII-like and ScrFI-like systems. Pre-methylation of pCP11 by pSS05 increased the conjugation efficiency in all the tested strains except in OSU THCO2-90, though the efficiencies varied widely. The low efficiencies observed for some strains may be due to the activity of additional strain-specific R-M systems (Fig. 1) that coexist with HpaII-like and ScrFI-like systems and may target other DNA motifs in pCP11. Intriguingly, although CN38 apparently lacks the two R-M systems, pSS05 enhanced conjugative DNA transfer into this strain. Perhaps the other R-M systems with unknown specificity that are present in this strain have similar functions.

Our *in vitro* experiments confirmed that pSS05 protected pCP11 from digestion by the REases HpaII and ScrFI. Growth of *E. coli* harboring pSS05 at low temperature was critical for the proper function of the M.FpsJI and M.FpsJVI MTases. *F. psychrophilum* requires temperatures of 4°C–23°C for growth (20, 21). *F. psychrophilum* MTases may be unstable at 37°C, a lethal temperature for *F. psychrophilum* but one that is often used for growth of *E. coli*. Although the donor *E. coli* was grown at 37°C for all conjugation experiments reported here, the conjugation process involved a 2-day incubation at 18°C, which should have allowed the *E. coli-*expressed M.FpsJI and M.FpsJVI to function properly and methylate the target DNA before conjugative transfer into *F. psychrophilum*. We do not know how much pre-methylation is needed for efficient protection of the DNA during conjugation. It is possible that a small amount of pre-methylation occurring during growth of *E. coli* at 37°C is sufficient to allow successful conjugation. However, incubation of *E. coli* at 18°C before conjugation might be important for optimal methylation and even more efficient DNA transfer.

We also investigated the role of M.FpsJIV system, despite the lack of a homologous gene encoding the cognate REase (MboI prototype). Indeed, it is one of the most prevalent MTases among the 17 analyzed genomes, and DNA methylation of cytosine residues in the predicted GATC motifs was confirmed by PacBio. Our results showed that pCP11 isolated from *E. coli* S17-1 λ *pir* without the M.FpsJIV methylation plasmid (pSS03) resisted digestion by MboI and pCP11 isolated from *E. coli* JM110, a strain lacking the DNA adenine methyltransferase (Dam), was digested by MboI (Fig. S3). We propose that Dam expressed from the chromosome of *E. coli* S17-1 λ *pir* methylates adenosine in the same GATC motifs and thus protects DNA from digestion by MboI (82). This methylation may be important for successful DNA transfer, but we did not test conjugation from *E. coli* strains that have been engineered to lack *dam* gene function.

The other tested MTases including M.FpsJII, M.FpsJIII, M.FpsJV, and M.FpsJVII may also be important. Unsuccessful conjugation by using the methylation plasmids carrying their encoding genes probably means methylation of these recognition motifs individually could not fully protect pCP11. It is also possible that these MTases were not expressed successfully or the expression levels were too low in the donor *E. coli*. To advance this methodology and protect more types of restriction sites simultaneously, an alternative strategy is to chemically synthesize a methylation plasmid that carries many *F. psychrophilum* MTase-encoding genes optimized for expression in *E. coli*. Customized promoters could be used to control the expression of these enzymes.

The methylation plasmid pSS05 protected two commonly used vectors (pCP11 and pYT313) for genetic analysis of *Flavobacterium* species during their conjugative transfer into *F. psychrophilum*. The use of pSS05 and pYT313 allowed us to delete the gene encoding the T9SS core component GldN in *F. psychrophilum* strain CSF259-93 and to efficiently construct other mutants in *F. psychrophilum* strains CSF259-93, CN06, and DIFR 950106-1/1 (unpublished results).

With the availability of these new tools, it is now possible to conduct genetic experiments to identify virulence factors of diverse *F. psychrophilum* strains, which may eventually lead to the development of live attenuated vaccines or other measures to prevent and control BCWD. In addition, a similar approach may allow or improve the efficiency of genetic manipulation in many other members of the phylum *Bacteroidota*, including important animal and human pathogens, and in many other bacteria for which such experiments have failed in the past.

## MATERIALS AND METHODS

### Bacterial strains, plasmids, and growth conditions

*F. psychrophilum* strains CSF259-93 (64, 65), CN06 (19, 83), CN38 (83), DIFR 950106-1/1 (66, 84), FI056 (42), FRGDSA 1882/11 (42), JIP08/99 (42), LM-01-Fp (42), LM-02-Fp (42), and OSU THCO2-90 (41) were the wild-type strains used in this study (Table S1). *F. psychrophilum* strains were grown at 18°C in tryptone yeast extract salt (TYES) medium (8, 85). TYES contains 4 g/L tryptone, 0.4 g/L yeast extract, 0.5 g/L MgSO_4_**·**7H_2_O, and 0.5 g/L CaCl_2_**·**2H_2_O, with pH adjusted to 7.2. For solid media, 15 g agar was added per liter unless indicated otherwise. *E. coli* strains were grown in lysogeny broth (LB) at 37°C (86, 87). For all experiments, *F. psychrophilum* strains were propagated from -80°C glycerol stocks on TYES agar and incubated for 72 h or until obvious growth was observed at 18°C before they were used as starter cultures. Strains and plasmids used in this study are listed in Table S1, and primers are listed in Table S2. Antibiotics were used at the following concentrations when needed: ampicillin, 100 µg/mL; chloramphenicol, 10 µg/mL; and erythromycin, 20 µg/mL.

### Construction of methylation plasmids

A 1.8 kbp fragment, spanning the CSF259-93 M.FpsJI-encoding gene, FPSM_02394, and its 450 bp upstream and 80 bp downstream regions, was amplified by PCR using Phusion DNA polymerase (Thermo Fisher Scientific, Waltham, MA) and primers 0090 (introducing a BamHI site) and 0091 (introducing a SalI site). The fragment was digested with BamHI and SalI and ligated into pACYC184 (75, 76) that had been digested with the same enzymes to generate pSS01. This process truncated and inactivated the pACYC184 *tetR* gene (Fig. 2). pSS02 carrying the M.FpsJVI-encoding gene FPSM_00612 and pSS03 carrying the M.FpsJIV-encoding gene FPSM_01581 were constructed using the same procedure with the appropriate primers listed in Table S2. To construct pSS05, which carries the M.FpsJVI- and M.FpsJI-encoding genes, FPSM_00612 was amplified with primers 0133 and 0134, digested by SalI and NarI, and ligated into SalI and NarI digested pSS01. To construct pSS07, which carries the M.FpsJII-encoding gene, FPSM_00552 was amplified with primers 0138 and 0139, digested by BamHI and NarI, and ligated into BamHI and NarI digested pSS01. The same procedure was used to construct pSS08 carrying the M.FpsIVII-encoding gene FPSM_00649, pSS11 carrying the M.FpsJV-encoding gene FPSM_01519, and pCB01 carrying the M.FpsJIII-encoding gene FPSM_01246 using the primers listed in Table S2.

### Conjugative transfer of plasmids into *F. psychrophilum* strains

Plasmids were transferred from *E. coli* S17-1 λ *pir* into *F. psychrophilum* strains by conjugation. The *E. coli/F. psychrophilum* shuttle vector pCP11 (74) was used as a reporter plasmid to test the efficiency of conjugations. Prior to conjugation, pCP11 was co-transformed with the appropriate methylation plasmids into *E. coli* S17-1 λ *pir*, generating the donor strains. pACYC184 was also co-transformed with pCP11 to use as a non-methylating negative control.

The *E. coli* donor strains were inoculated into LB with appropriate antibiotics and incubated with shaking for 16 h at 37°C. Fresh *F. psychrophilum* starter cultures were lawn inoculated onto TYES agar and incubated at 18°C for 40–48 h. The *E. coli* cells were collected by centrifugation (4,000 × *g*, 10 min, 20°C) and washed once with TYES broth. The *F. psychrophilum* cells were scraped off of the plates, suspended in TYES broth, centrifuged (4,000 × *g*, 10 min, 20°C), and washed once with TYES broth. All the cell pellets were resuspended in TYES, and their OD_600_ was measured using a microplate reader (Thermo Scientific Multiskan Spectrum) with 200 µL of sample loaded in each well of the flat bottom 96-well plates. The amounts of cells used in conjugations were quantified by OD_600_ × volume (µL). Equal amounts of *F. psychrophilum* and *E. coli* cells (OD_600_ × volume ≈ 2,200) were mixed, concentrated to approximately 100 µL, spotted on antibiotic-free TYES plates, dried until the liquid was invisible, and incubated at 18°C for 2 days. After conjugation, each spot of cell mixture was scraped off from the agar and suspended in 1 mL TYES. One hundred microliters of undiluted, 10-fold diluted, and 100-fold diluted cell suspensions were plated onto TYES agar with 20 µg/mL of erythromycin and incubated at 18°C for 7–10 days. Colonies grown on each plate were counted after incubation, and all the numbers were standardized to CFUs / plate with 100 µL undiluted cell suspension. Colonies were randomly selected to confirm the presence of pCP11 by PCR.

### *In vitro* restriction digestion of pCP11

Strains of *E. coli* S17-1 λ *pir* harboring pCP11, pSS05, or pCP11and pSS05 were streaked from -80°C onto LB agar plate with appropriate antibiotics. After incubation overnight at 37°C, one corresponding single isolated colony was inoculated onto LB with antibiotics and shaken overnight at 37°C. The plasmids were extracted by using the IBI Scientific Mini High-Speed Plasmid kit. To incubate *E. coli* cells at 18°C, a cell pellet from 1 mL overnight culture grown at 37°C was transferred into fresh LB with proper antibiotics and subsequently cultured at 18°C for 18 h. pCP11 extracted from *F. psychrophilum* CSF259-93 grown at 18°C was used as a control. Five hundred ng plasmid DNA was used for each of the 50 µL digestion reaction assay. Ten units of HpaII or five units of ScrFI (New England Biolabs, Ipswich, MA) were used in each reaction. DNA electrophoresis with 0.8% agarose gel containing GelRed gel stain was performed to obtain gel images.

### Construction of deletion mutants lacking the Fps.HpaII, Fps.ScrFI, and GldN encoding genes

To construct the deletion plasmids for the *F. psychrophilum* CSF259-93 Fps.HpaII and Fps.ScrFI encoding genes (FPSM_02393 and FPSM_00613, respectively), the ClonExpress Ultra One Step Cloning Kit (Vazyme, Nanjing) was used by multiple-fragment homologous recombination. BamHI and SalI were selected as the digestion sites to linearize the suicide vector pYT313 (47). Primers for amplification of the inserted fragments were automatically generated using Vazyme’s primer design software, CE Design (http://www.vazyme.com). An insert fragment of 1,699 bp spanning the first 99 bp of FPSM_00613 and its upstream region was amplified using primers 0293 and 0294. A second fragment of 1,252 bp spanning the last 99 bp of FPSM_00613 and its downstream region was amplified using primers 0295 and 0296 (Fig. S2). The two fragments were proportionally mixed with the linearized pYT313 and assembled under the recombinase (Exnase) catalyzed reaction, according to the manufacturer’s instructions, to generate the deletion plasmid pXG01. The same method was used to construct pXG02 for deletion of FPSM_02393, using the primers listed in Table S2.

To delete *gldN* in *F. psychrophilum* CSF259-93, a 2,058 bp fragment spanning *gldM* and the first 84 bp of *gldN* was amplified using primers 0117 (introducing a BamHI site) and 0118 (introducing a SalI site) (Fig. S2). The fragment was digested with BamHI and SalI and ligated into pYT313, which had been digested with the same enzymes, to generate pSS04. A 2,036 bp fragment spanning FPSM_00827, FPSM_00828, and the final 36 bp of *gldN* was amplified with primers 0119 (introducing a SalI site) and 0120 (introducing an SphI site). The fragment was cloned between the SalI and SphI sites of pSS04 to generate the deletion construct pSS12.

Each of the deletion plasmids (pSS12, pXG01, and pXG02) was co-transformed with pSS05 into *E. coli* S17-1 λ *pir* and introduced into *F. psychrophilum* CSF259-93 by conjugation as described above. Selection for erythromycin resistance resulted in colonies that had the deletion plasmid integrated into the chromosome by homologous recombination. Colonies were streaked on erythromycin plates for isolation. The cells from an isolated colony were inoculated into 3 mL of TYES medium without antibiotics and incubated at 18°C with shaking for 24 h to allow plasmid excision by a second recombination event. These cells were serially diluted and plated on TYES agar containing 2.5% sucrose and incubated at 18°C until isolated colonies appeared. The sucrose-resistant colonies were patched in replica on sucrose plates and erythromycin plates. Colonies that grew on sucrose but not on erythromycin were streaked for isolation and screened by PCR using primers 0195 and 0196, which flank the *gldN* coding sequence (Fig. S2), to obtain the *gldN* deletion mutants. Primers upstream of primer 0117 and downstream of primers 0120, 0391, and 0392, respectively, were used to amplify the region spanning the *gldN* deletion from chromosomal DNA of the mutant. The PCR product was sequenced, confirming that the desired mutation was obtained. Primers used for screening and sequencing of the FPSM_02393 and FPSM_00613 deletion mutants are listed in Table S2.

### Complementation of *gldN* mutant

Primers 0195 (introducing a KpnI site) and 0196 (introducing an SphI site) were used to amplify a 1,306 bp fragment spanning *gldN* from *F. psychrophilum* CSF259-93 genomic DNA (Fig. S2). The fragment was digested with KpnI and SphI and ligated into pCP11 that had been digested with the same enzymes to generate pSS13. The plasmid was co-transformed with pSS05 into *E. coli* S17-1 λ *pir* and transferred to the *gldN* mutant by conjugation.

### Analysis of colony spreading and cell motility

*F. psychrophilum* CSF259-93 wild type, Δ*gldN* mutant, and the complemented strain were streaked on 5% TYES (TYES diluted 20-fold with distilled water) solidified with 1% agar (46). The plates were incubated for 8 days at 18°C. Isolated colonies were examined using a Moment CMOS camera (Teledyne Photometrics, Tucson, AZ) mounted on a Nikon Eclipse TS100 inverted microscope. Motility of individual cells on agar was examined by spotting cells (grown in TYES broth for 48 h with shaking at 18°C) on a pad of full-strength TYES solidified with 1% agar on a glass slide, allowing the spot to dry briefly, and covering it with an O_2_-permeable Teflon membrane (Yellow Springs Instrument Co., Yellow Springs, OH) that prevented dehydration and served as a coverslip (46). Cell movements over agar were observed using a Nikon Ci-L plus phase-contrast microscope at room temperature (22°C). Images were recorded and analyzed using an SC2000C CMOS camera and CapStudio (Image Technology, Suzhou, China). Rainbow traces of cell movements were made using Fiji (https://imagej.net/) and the macro Color FootPrint (88).

### Analysis of proteolytic activity

Cell cultures of *F. psychrophilum* CSF259-93 wild type, Δ*gldN*, and the complemented strain were grown at 18°C for 24 h and adjusted to OD_600_ = 0.5. Three microliters of each culture was spotted on TYES agar supplemented with 1.5% of skim milk (Thermo Fisher). To prepare the media, the 2× TYES agar and 3% skim milk stock solution in water were autoclaved separately and then combined. Proteolytic activity was observed on the plates with clearing of precipitated protein surrounding the area of growth. The plates were incubated at 18°C for 7 days, and pictures were obtained using a Bio-Rad imaging system under visible light.

### Rainbow trout genetic lines, rearing conditions, and water quality parameters

Rainbow trout were obtained as eyed-eggs (Troutlodge Inc, WA) and reared under standard feed conditions with 13°C flow-through spring water with dissolved oxygen at or above 12 mg/L. The genetic stock of fish in trial 1 was from the Troutlodge February spawning line, and fish had a mean body weight (bwt) of 2.7 g at the time of challenge (age 72 days post-hatch). In trial 2, Troutlodge May spawning line was used, and fish were 14.2 g bwt at the time of challenge (age 127 days post-hatch).

### Standardized laboratory challenge

*F. psychrophilum* strains were grown and prepared as described (89). Briefly, a dilution of an -80°C frozen stock was plated on TYES plates at 15°C for 5 days and the harvested bacteria resuspended in Dulbecco’s PBS (Sigma). Cell concentration was measured by optical density of the suspension at 525 nm. Bacterial cell number was verified by direct plate counting of triplicate cultures. Fish were anesthetized with 100 mg/L tricaine methanesulfonate (Tricaine-S, Western Chemical, Inc., Ferndale, WA) prior to challenge. Fish were challenged by intramuscular injection in the mid-point between the start of the dorsal fin and lateral line using a 26 g needle attached to an Eppendorf repeating syringe. Randomly assigned, triplicate tanks were used for all treatments with either 38–41 fish per tank in trial 1 and 19–20 fish per tank in trial 2. In trial 1, each fish was injected with 20 µL bacterial suspension, while in trial 2, each fish was injected with 50 µL bacterial suspension. Control fish were injected in the same location with the corresponding volume of sterile PBS per experiment. Moribund or dead fish were removed daily between the times of 7:30 am and 9:30 am for 28 days. Fish were fed twice daily prior to challenge and not fed the day of challenge. Fish were fed to satiation once per day during the challenge.

### Bioinformatic analyses

Genome and gene sequences of *F. psychrophilum* CSF259-93 were obtained from IMG (https://img.jgi.doe.gov/). All primers, plasmid maps, and gene maps were generated using MacVector (Version 18.2.5). The Restriction Enzyme REBASE database (http://rebase.neb.com/rebase/rebase.html) (69) was used to analyze the R-M systems present in *F. psychrophilum* CSF259-93.

Comparison of the DNA methyltransferase gene content between strains was performed on a selection of 17 publicly available genomes (42, 65, 73, 90). Sequences were retrieved from NCBI and EMBL databases using the following accession numbers: JIP02/86 (AM398681.2), CSF259-93 (CP007627), DIFR 950106-1/1 (CP008902.1), JIP08/99 (GCA_900186595), LM-02-Fp (GCA_900186665), IT09 (GCA_900186525), CN06 (NZ_CP046374.1), FI056 (GCA_900186375), DK002 (GCA_900186425), LM-01-Fp (GCA_900186685), IT02 (GCA_900186545), FRGDSA 1882/11 (GCA_900186415), CN38 (NZ_CP081494.1), FPC840 (GCA_900186575), KU060626-59 (GCA_900186495), NCIMB 1947T (CP007207), and OSU THCO2-90 (LT670843). The putative DNA MTase-encoding genes were identified using the web interface MicroScope (91) and were manually curated and classified in type I, II, IIG, and III systems using the REBASE database (69). Orthologs were identified using BlastP Bidirectional Best Hit.

## Data Availability

The strains and plasmids are available from the authors upon request. All data associated with this work are included in the article and its supplemental material.

## SUPPLEMENTAL MATERIAL

Supplemental material aem.01448-24-s0001.pdf Tables S1 and S2; Figures S1 to S3.

ASM does not own the copyrights to Supplemental Material that may be linked to, or accessed through, an article. The authors have granted ASM a non-exclusive, world-wide license to publish the Supplemental Material files. Please contact the corresponding author directly for reuse.
