# Supplementary material for: Methylation of foreign DNA overcomes the restriction barrier of Flavobacterium psychrophilum and allows efficient genetic manipulation
Source: Appl Environ Microbiol. 2025 Jan 10;91(2):e01448-24. doi: 10.1128/aem.01448-24 (PMC11837570; doi:10.1128/aem.01448-24)
Supplement: Supplemental material — Tables S1 and S2; Figures S1 to S3. [file aem.01448-24-s0001.pdf]

Supplemental Tables and Figures for AEM01448-24:

Methylation of foreign DNA overcomes the restriction barrier of *Flavobacterium psychrophilum* and allows efficient genetic manipulation

Seada Sloboda, Xinwei Ge, Daqing Jiang, Lin Su, Gregory D. Wiens, Carly A. Beveridge, Eric Duchaud, Mark J. McBride, Tatiana Rochat, and Yongtao Zhu

Table S1 Strains and plasmids used in this study.

Strain or plasmid	Description[a]	Source or reference
E. coli strains		
DH5αMCR	Strain used for cloning	Life Technologies (Grand Island, NY)
DH5α	Strain used for cloning	Sangon Biotech (Shanghai, China)
S17-1 λ *pir*	Strain used for conjugation	(1)
JM110	Strain used for preparing plasmid DNA free of Dam methylation	Weidi Biotechnology (Shanghai, China)
F. psychrophilum strains		
CN06	Wild type; isolated from skin ulcer of *O. mykiss* in Gansu, China; CC-ST10	(2, 3)

CN38	Wild type; isolated from *O. mykiss* in Liaoning, China	(3)
CSF259-93	Wild type; isolated from spleen of *O. mykiss* in Idaho, United States; CC-ST10	(4, 5)
DIFR 950106-1/1	Wild type; isolated from *O. mykiss* in Denmark; CC-ST10	(6, 7)
FI056	Wild type; isolated from inner organs of *O. mykiss* in Finland; CC-ST10	(8)
FRGDSA 1882/11	Wild type; isolated from *O. mykiss* in France; CC-ST90	(8)
JIP08/99	Wild type; isolated from kidney of *O. mykiss* in France; CC-ST10	(8)
LM-01-Fp	Wild type; isolated from kidney of *O. mykiss* in Chile; CC-ST10	(8)
LM-02-Fp	Wild type; isolated from kidney of *O. mykiss* in Chile; CC-ST10	(8)
OSU THCO2-90	Wild type; isolated from kidney of *O. kisutch* in Oregon, United States; CC-ST9	(9)
FpC8	Δ*gldN* (FPSM_00826) in CSF259-93	This study
FpC74	Δ*Fps.ScrFI* (FPSM_00613) in CSF259-93	This study
FpC75	Δ*Fps.HpaII* (FPSM_02393) in CSF259-93	This study
Plasmids		

pACYC184	p15A *ori*; Cm^r Tc^r; plasmid for cloning of the MTase-encoding genes	ATCC, (10, 11)
pCP11	pMB1 *ori*; (pCP1 *ori*); Ap^r $(Em)^r$; *E. coli*-*F. psychrophilum* shuttle plasmid	(12)
pCP23	pMB1 *ori*; (pCP1 *ori*); Ap^r $(Tc)^r$; *E. coli*-*F. psychrophilum* shuttle plasmid	(13)
pYT313	pMB1 *ori*; Ap^r $(Em)^r$; suicide vector carrying *sacB* used to make chromosomal deletions	(14)
pSS01	Pre-methylation plasmid containing CSF259-93 M.FpsJI-encoding gene FPSM_02394, amplified with primers 0090/0091 and cloned between the BamHI/SalI sites of pACYC184; Cm^r	This study
pSS02	Pre-methylation plasmid containing CSF259-93 M.FpsJVI-encoding gene FPSM_00612, amplified with primers 0092/0093 and cloned between the BamHI/SalI sites of pACYC184; Cm^r	This study
pSS03	Pre-methylation plasmid containing CSF259-93 M.FpsJIV-encoding gene FPSM_01581, amplified with primers 0094/0095 and cloned between the BamHI/SalI sites of pACYC184; Cm^r	This study
pSS04	2.1 kbp region upstream of CSF259-93 *gldN* (FPSM_00826) amplified with primers 0117/0118 and	This study

	cloned between the BamHI/SalI sites of pYT313; Ap^{r} (Em^{r})	
pSS05	Pre-methylation plasmid containing CSF259-93 M.FpsJI and M.FpsJVI-encoding genes; FPSM_00612 was amplified with primers 0133/0134 and cloned between the SalI/NarI sites of pSS01; Cm^{r}	This study
pSS07	Pre-methylation plasmid containing CSF259-93 M.FpsJII-encoding gene FPSM_00552, amplified with primers 0138/0139 and cloned between the BamHI/NarI sites of pSS01; Cm^{r}	This study
pSS08	Pre-methylation plasmid containing CSF259-93 M.FpsJVII-encoding gene FPSM_00649, amplified with primers 0140/0141 and cloned between the BamHI/NarI sites of pSS01; Cm^{r}	This study
pSS11	Pre-methylation plasmid containing CSF259-93 M.FpsJV-encoding gene FPSM_01519, amplified with primers 0145/0146 and cloned between the BamHI/NarI sites of pSS01; Cm^{r}	This study
pSS12	Construct used to delete *gldN* in CSF259-93; 2.0 kbp region downstream of *gldN* amplified with primers 0119/0120 and cloned between the SalI/SphI sites of pSS04; Ap^{r} (Em^{r})	This study

pSS13	Plasmid for complementation of *gldN* in CSF259-93; *gldN* was amplified with primers 0195/0196 and cloned between the KpnI/SphI sites of pCP11; Ap^r (Em^r)	This study
pCB01	Pre-methylation plasmid containing CSF259-93 M.FpsJIII-encoding gene FPSM_01246, amplified with primers 0142/0143 and cloned between the BamHI/NarI sites of pSS01; Cm^r	This study
pXG01	Construct used to delete the Fps.ScrFI-encoding gene FPSM_00613 in CSF259-93; 1.7 kbp region upstream of FPSM_00613 amplified with primers 0293/0294 and 1.3 kbp region downstream of FPSM_00613 amplified with primers 0295/0296 cloned between the SalI/BamHI sites of pYT313; Ap^r (Em^r)	This study
pXG02	Construct used to delete the Fps.HpaII-encoding gene FPSM_02393 in CSF259-93; 1.7 kbp region upstream of FPSM_02393 amplified with primers 0297/0298 and 1.7 kbp region downstream of FPSM_02393 amplified with primers 0299/0300 cloned between the SalI/BamHI sites of pYT313; Ap^r (Em^r)	This study

[a]Antibiotic resistance phenotypes: ampicillin-Ap^r, chloramphenicol-Cm^r, erythromycin-Em^r, and tetracycline-Tc^r. Unless indicated otherwise, the antibiotic resistance phenotypes are those

expressed in *E. coli*. The antibiotic resistance phenotypes given in parentheses are those expressed in *F. psychrophilum* but not in *E. coli*. p15A or pMB1 *ori* functions in *E. coli* but not in *F. psychrophilum.* pCP1 *ori* functions in *F. psychrophilum* but not in *E. coli*.

Table S2 Primers used in this study.

Primers	Sequence and Description
0090	5' GCTAGGGATCCTATTATTGGGGTTAGAGGAATC 3'; forward primer used in construction of pSS01; BamHI site underlined
0091	5' GCTAGGTCGACTATTCAAATTGGCATCTCCA 3'; reverse primer used in construction of pSS01; SalI site underlined
0092	5' GCTAGGGATCCAGCAGGGAAAAGTTTTGAAG 3'; forward primer used in construction of pSS02; BamHI site underlined
0093	5' GCTAGGTCGACGAAGTGGCGGATTTAGAA 3'; reverse primer used in construction of pSS02; SalI site underlined
0094	5' GCTAGGGATCCACCGTATCAAAGACGATTTT 3'; forward primer used in construction of pSS03; BamHI site underlined
0095	5' GCTAGGTCGACACCTTAGAAGATTTTCAATATGC 3'; reverse primer used in construction of pSS03; SalI site underlined
0117	5' GCTAGGGATCCGATGCAATGTTAAAAGAGGC 3'; forward primer used in construction of pSS04; BamHI site underlined
0118	5' GCTAGGTCGACAGCATTCAACAAGTTAGATTG 3'; reverse primer used in construction of pSS04; SalI site underlined
0119	5' GCTAGGTCGACGTTCGTAATTTCGAGCAAGA 3'; forward primer used in construction of pSS12; SalI site underlined
0120	5' GCTAGGCATGCCGGTTCTTCCTTCTTCAAAA 3'; reverse primer used in construction of pSS12; SphI site underlined
0133	5' GCTAGGTCGACAGCAGGGAAAAGTTTTGAAG 3'; forward primer used in construction of pSS05; SalI site underlined
0134	5' GCTAGGGCGCCGAAGTGGCGGATTTAGAA 3'; reverse primer used in construction of pSS05; NarI site underlined
0138	5' GCTAGGGATCCCCAATACTTCCCTAATGCAA 3'; forward primer used in construction of pSS07; BamHI site underlined
0139	5' GCTAGGGCGCCTGCTGCGGTGTATAATATAA 3'; reverse primer used in construction of pSS07; NarI site underlined
0140	5' GCTAGGGATCCAGCGCACTTAGAAACTTAAA 3'; forward primer used in construction of pSS08; BamHI site underlined
0141	5' GCTAGGGCGCCTACAATCCAAGCCAATCATT 3'; reverse primer used in construction of pSS08; NarI site underlined
0142	5' GCTAGGGATCCTGGGTTTCGATTTATAGAGC 3'; forward primer used in construction of pCB01; BamHI site underlined
0143	5' GCTAGGGCGCCGCCCACTTTAATACAAAAGC 3'; reverse primer used in construction of pCB01; NarI site underlined
0145	5' GCTAGGGATCCATTTAGCCAAAAATTGAGCG 3'; forward primer used in construction of pSS11; BamHI site underlined
0146	5' GCTAGGGCGCCATTGTTTCAAGTCCGTTTTT 3'; reverse primer used in construction of pSS11; NarI site underlined
0195	5' GCTAGGGTACCTAGTTGTTTCTGGAAGCAAA 3'; forward primer used in construction of pSS13; KpnI site underlined

0196	5' GCTAGGCATGCCGGAGCCAATAATTAGGTAG 3'; reverse primer used in construction of pSS13; SphI site underlined
0293	5' cttgcatgcctgcagGTCGACAATAACTCCATAAAATTCACGCTACAA 3'; forward primer used in construction of pXG01; SalI site underlined
0294	5' agcgactgaAATATATTTAGTCCTGTATTGTTTGATTAATGT 3'; reverse primer used in construction of pXG01
0295	5' ctaaatatattTCAGTCGCTTTAAAATCAATGAAAA 3'; forward primer used in construction of pXG01
0296	5' gcggaaaaattcgggGGATCCAGGTGTAAAAATCTCAAAAGTGTGAAG 3'; reverse primer used in construction of pXG01; BamHI site underlined
0297	5' cttgcatgcctgcagGTCGACAGAAGAAATAGGTACTGGTGGTGGC 3'; forward primer used in construction of pXG02; SalI site underlined
0298	5' gaactacttgcagtttctaaTTTATTCAAATTGGCATCTCCAGC 3'; reverse primer used in construction of pXG02
0299	5' TTAGAAACTGCAAGTAGTTCAAGACACG 3'; forward primer used in construction of pXG02
0300	5' gcggaaaaattcgggGGATCCTCATACAAATGATTAAATACGTTATTGTTTT 3'; reverse primer used in construction of pXG02; BamHI site underlined
0334	5' GCTAGGGTACCGCAAACAGCTTACTTGGCGT 3'; forward primer used in screening of the FPSM_00613 (Fps.ScrFI) deletion mutant
0335	5' GCTAGGCATGCCGTGTTCAGGAATTTGGGT 3'; reverse primer used in screening of the FPSM_00613 (Fps.ScrFI) deletion mutant
0336	5' GCTAGGGTACCTTGGGTTTCGTCAGGATT 3'; forward primer used in screening of the FPSM_02393 (Fps.HpaII) deletion mutant
0337	5' GCTAGGCATGCTCGAAAAACCTCCTGATGTTGG 3'; reverse primer used in screening of the FPSM_02393 (Fps.HpaII) deletion mutant
0338	5' ATTATTTAGAAGTAGTTTACTTGATG 3'; forward primer used to sequence the FPSM_00613 (ScrFI) deletion mutant
0339	5' CTCCGCGAAAGTTACCACCAAC 3'; reverse primer used to sequence the FPSM_00613 (ScrFI) deletion mutant
0340	5' GCTCCCGCACCTATTATTGGGG 3'; forward primer used to sequence the FPSM_02393 (HpaII) deletion mutant
0341	5' GGCACCAAATCCTGAAAATAGCGC 3'; reverse primer used to sequence the FPSM_02393 (HpaII) deletion mutant
0391	5' TAGGGTTATTAACTGAGGCA 3'; forward primer used to sequence the *gldN* deletion mutant
0392	5' TTCGCTCTCATAATCTGTTC 3'; reverse primer used to sequence the *gldN* deletion mutant

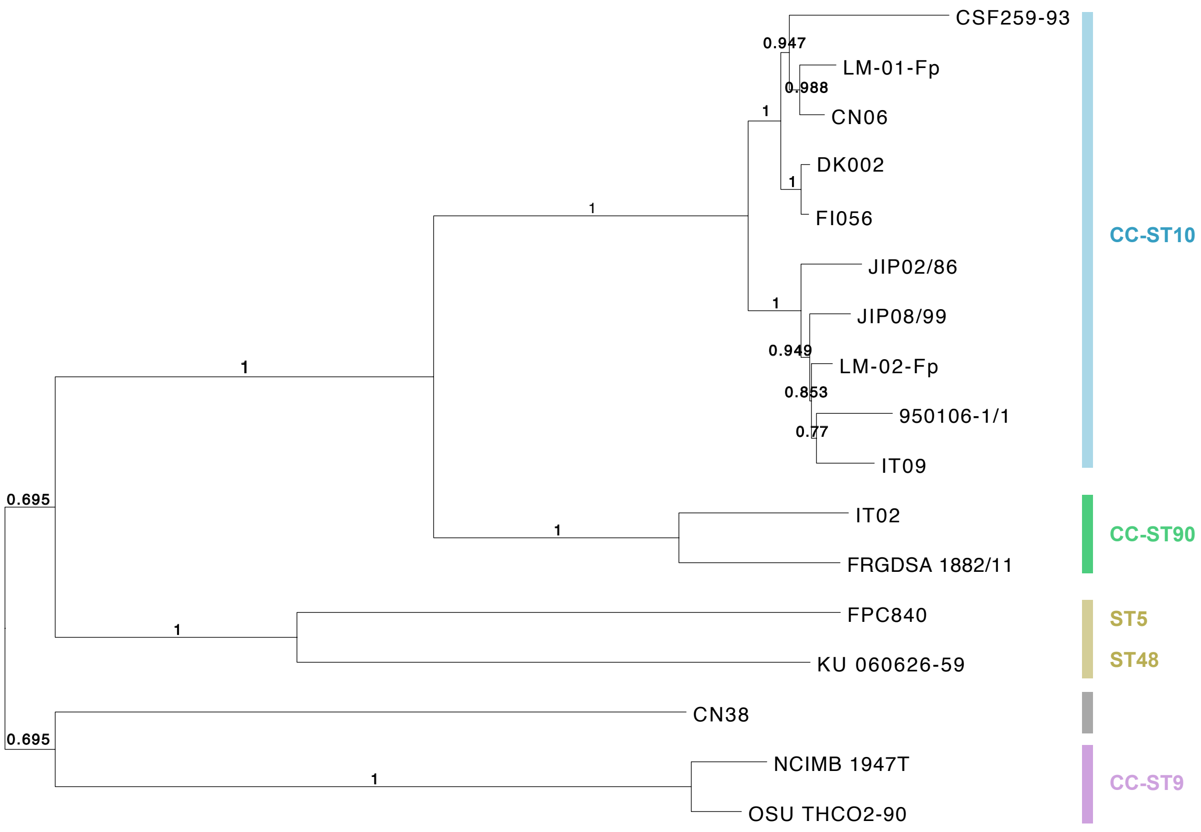

Figure S1 Phylogenomic core proteins tree of *F. psychrophilum* strains. MicroScope platform (15) was used to extract sequences of core proteins (*i.e.*, conserved and ubiquitous proteins encoded by core genome genes). A cutoff of 80% identity and 80% on the minimal coverage of the length between the aligned portions of two proteins was chosen to determine whether two CDSs were members of the same gene family. 1913 core proteins were retrieved and each individual alignment was concatenated using an in-house R script, as previously reported (16). Core proteome tentative phylogenetic tree reconstruction was performed after Gblocks 0.91.1 curation using FastTree 2.1.11 reconstruction and the NGPhylogeny.fr package (17) with default parameters. Transfer bootstrap expectation values (18) are indicated at branch nodes.

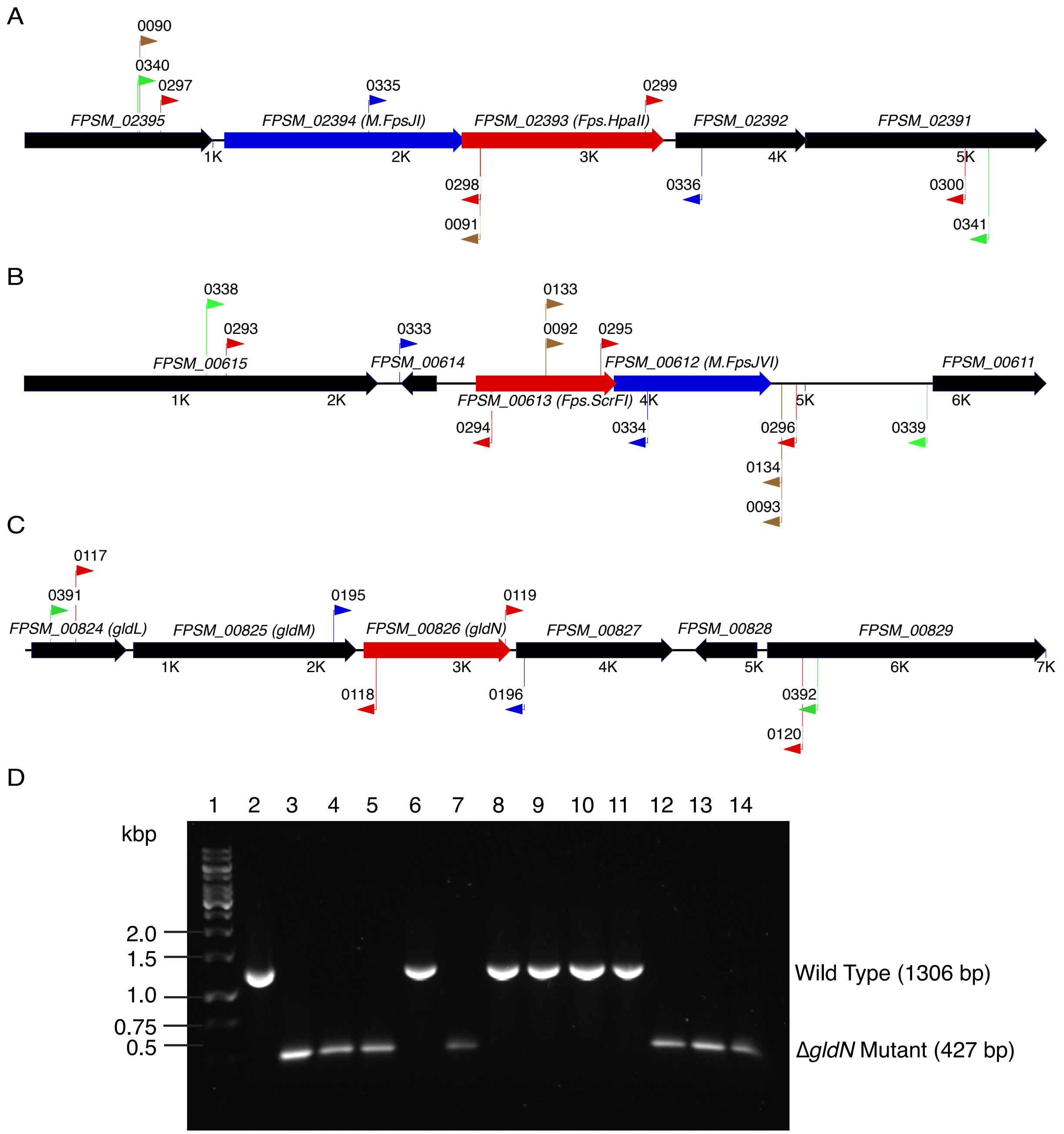

Figure S2 Maps of the *F. psychrophilum* CSF259-93 HpaII-M.FpsJI (A), ScrFI-M.FpsJVI (B), and *gldN* (C) regions, and PCR confirmation of *gldN* deletion in CSF259-93 (D). Numbers below the maps refer to kilobase pairs of sequence. Binding sites for primers used in PCR reactions to clone the MTases (brown arrows), generate deletion (red arrows), screen the deletion mutants (blue arrows), or sequence the deletion regions (green arrows) are shown above

and below the maps. Primers 0195 and 0196 were used in PCR in Panel D. 1, DNA ladder (Thermo Scientific™, SM1163); 2, WT control; 3, pSS12 (*gldN* deletion plasmid) control; 4-14, colonies grown on sucrose plates after the second recombination. Colonies in lanes 6, 8, 9, 10, and 11 are wild type and colonies in lanes 4, 5, 7, 12, 13, and 14 are *gldN* deletion mutants. Similar results were obtained for the deletion of Fps.HpaII and Fps.ScrFI encoding genes (data not shown).

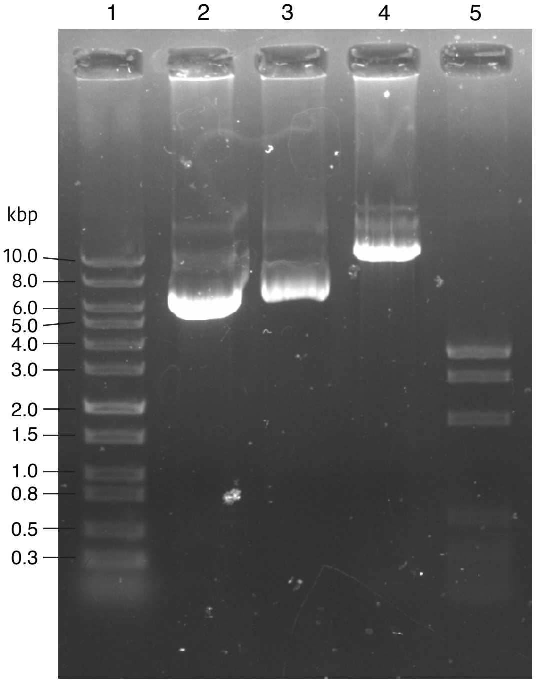

Figure S3 *In vitro* restriction digestion of pCP11 by MboI. Lane 1, DNA ladder (TransGene Biotech, BM211); lane 2, pCP11 isolated from *E. coli* S17-1 λ *pir*; lane 3, pCP11 isolated from *E. coli* S17-1 λ *pir* and digested by MboI; lane 4, pCP11 isolated from *E. coli* JM110, a strain lacking the DNA adenine methyltransferase (Dam); lane 5, pCP11 isolated from *E. coli* JM110 and digested by MboI. Approximately 300 ng DNA digested with 0.6 U of MboI was loaded in lane 3 or lane 5. The *E. coli* strains contained pCP11 were cultivated at 37°C.

References

1. Simon R, Priefer U, Pühler A. 1983. A Broad Host Range Mobilization System for In Vivo Genetic Engineering: Transposon Mutagenesis in Gram Negative Bacteria. Nat Biotechnol 1:784-791.
2. Li S, Chai J, Knupp C, Nicolas P, Wang D, Cao Y, Deng F, Chen F, Lu T, Loch Thomas P. 2021. Phenotypic and Genetic Characterization of *Flavobacterium psychrophilum* Recovered from Diseased Salmonids in China. Microbiol Spectr 9:e00330-21.
3. Chen F, Wang D, Lu T, Li S. 2023. Identification of a novel type II-C Cas9 from the fish pathogen *Flavobacterium psychrophilum*. Front Microbiol 14:1181303.
4. Sudheesh PS, LaFrentz Br Fau - Call DR, Call Dr Fau - Siems WF, Siems Wf Fau - LaPatra SE, LaPatra Se Fau - Wiens GD, Wiens Gd Fau - Cain KD, Cain KD. 2007. Identification of potential vaccine target antigens by immunoproteomic analysis of a virulent and a non-virulent strain of the fish pathogen *Flavobacterium psychrophilum*. Dis Aquat Organ 74:37-47.
5. Wiens GD, LaPatra SE, Welch TJ, Rexroad C, 3rd, Call DR, Cain KD, LaFrentz BR, Vaisvil B, Schmitt DP, Kapatral V. 2014. Complete Genome Sequence of *Flavobacterium psychrophilum* Strain CSF259-93, Used To Select Rainbow Trout for Increased Genetic Resistance against Bacterial Cold Water Disease. Genome Announc 2:e00889-14.
6. Stenholm AR, Dalsgaard I Fau - Middelboe M, Middelboe M. 2008. Isolation and characterization of bacteriophages infecting the fish pathogen *Flavobacterium psychrophilum*. Appl Environ Microbiol 74:4070-4078.
7. Cisar JO, Bush CA, Wiens GD. 2019. Comparative Structural and Antigenic Characterization of Genetically Distinct *Flavobacterium psychrophilum* O-Polysaccharides. Front Microbiol 10:1041.
8. Duchaud E, Rochat T, Habib C, Barbier P, Loux V, Guérin C, Dalsgaard I, Madsen L, Nilsen H, Sundell K, Wiklund T, Strepparava N, Wahli T, Caburlotto G, Manfrin A, Wiens GD, Fujiwara-Nagata E, Avendaño-Herrera R, Bernardet JF, Nicolas P. 2018. Genomic Diversity and Evolution of the Fish Pathogen *Flavobacterium psychrophilum*. Front Microbiol 9.
9. Rochat T, Barbier P, Nicolas P, Loux V, Pérez-Pascual D, Guijarro JA, Bernardet JF, Duchaud EA-O. 2017. Complete Genome Sequence of *Flavobacterium psychrophilum* Strain OSU THCO2-90, Used for Functional Genetic Analysis Genome Announc 5:e01665-16.
10. Chang AC, Cohen SN. 1978. Construction and characterization of amplifiable multicopy DNA cloning vehicles derived from the P15A cryptic miniplasmid. J Bacteriol 134:1141-1156.
11. Rose RE. 1988. The nucleotide sequence of pACYC184. Nucleic Acids Res 16:355.
12. McBride MJ, Kempf MJ. 1996. Development of techniques for the genetic manipulation of the gliding bacterium *Cytophaga johnsonae*. J Bacteriol 178:583-590.
13. Agarwal S, Hunnicutt DW, McBride MJ. 1997. Cloning and characterization of the *Flavobacterium johnsoniae* (*Cytophaga johnsonae*) gliding motility gene, *gldA*. Proc Natl Acad Sci USA 94:12139-12144.
14. Zhu Y, Thomas F, Larocque R, Li N, Duffieux D, Cladière L, Souchaud F, Michel G, McBride MJ. 2017. Genetic analyses unravel the crucial role of a horizontally acquired

alginate lyase for brown algal biomass degradation by *Zobellia galactanivorans*. Environ Microbiol 19:2164-2181.
15. Vallenet D, Calteau A, Cruveiller S, Gachet M, Lajus A, Josso A, Mercier J, Renaux A, Rollin J, Rouy Z, Roche D, Scarpelli C, Médigue C. 2017. MicroScope in 2017: an expanding and evolving integrated resource for community expertise of microbial genomes. Nucleic Acids Res 45:D517-d528.
16. Bridel S, Bourgeon F, Marie A, Saulnier D, Pasek S, Nicolas P, Bernardet JF, Duchaud E. 2020. Genetic diversity and population structure of *Tenacibaculum maritimum*, a serious bacterial pathogen of marine fish: from genome comparisons to high throughput MALDI-TOF typing. Vet Res 51:60.
17. Lemoine F, Correia D, Lefort V, Doppelt-Azeroual O, Mareuil F, Cohen-Boulakia S, Gascuel O. 2019. NGPhylogeny.fr: new generation phylogenetic services for non-specialists. Nucleic Acids Res 47:W260-w265.
18. Lemoine F, Domelevo Entfellner JB, Wilkinson E, Correia D, Dávila Felipe M, De Oliveira T, Gascuel O. 2018. Renewing Felsenstein's phylogenetic bootstrap in the era of big data. Nature 556:452-456.
